# New directions in childhood obesity research: how a comprehensive biorepository will allow better prediction of outcomes

**DOI:** 10.1186/1471-2288-10-100

**Published:** 2010-10-22

**Authors:** Matthew A Sabin, Susan L Clemens, Richard Saffery, Zoe McCallum, Michele W Campbell, Wieland Kiess, Nancy A Crimmins, Jessica G Woo, Gary M Leong, George A Werther, Obioha C Ukoumunne, Melissa A Wake

**Affiliations:** 1 Murdoch Childrens Research Institute and Royal Children's Hospital, Flemington Road, Parkville, Victoria 3052, Australia; 2 University of Melbourne, Melbourne, Victoria 3010, Australia; 3 Hospital for Children and Adolescents, University of Leipzig, Liebigstr. 20a, D-04103 Leipzig, Germany; 4 Cincinnati Children's Hospital Medical Center, University of Cincinnati, USA; 5 The University of Queensland, Obesity Research Centre, Institute for Molecular Bioscience, St Lucia Queensland 4069, Australia; 6 Mater Children's Hospital, South Brisbane, Queensland 4010, Australia

## Abstract

**Background:**

Childhood obesity is associated with the early development of diseases such as type 2 diabetes and cardiovascular disease. Unfortunately, to date, traditional methods of research have failed to identify effective prevention and treatment strategies, and large numbers of children and adolescents continue to be at high risk of developing weight-related disease.

**Aim:**

To establish a unique 'biorepository' of data and biological samples from overweight and obese children, in order to investigate the complex 'gene × environment' interactions that govern disease risk.

**Methods:**

The 'Childhood Overweight BioRepository of Australia' collects baseline environmental, clinical and anthropometric data, alongside storage of blood samples for genetic, metabolic and hormonal profiles. Opportunities for longitudinal data collection have also been incorporated into the study design. National and international harmonisation of data and sample collection will achieve required statistical power.

**Results:**

Ethical approval in the parent site has been obtained and early data indicate a high response rate among eligible participants (71%) with a high level of compliance for comprehensive data collection (range 56% to 97% for individual study components). Multi-site ethical approval is now underway.

**Conclusions:**

In time, it is anticipated that this comprehensive approach to data collection will allow early identification of individuals most susceptible to disease, as well as facilitating refinement of prevention and treatment programs.

## Background

Obesity in early life is associated with many adverse effects on health including an increased risk of type 2 diabetes (T2DM), heart and liver disease [1]. In the majority of cases, weight gain is attributable to lifestyle-related factors [2], although a strong genetic contribution to body weight regulation is also recognised [3,4]. Since the first rare single-gene mutations were identified in severe early-onset obesity, far more common variants in key genes (and/or their promoter sequences) have been identified which explain significant weight variability within the population [5]. Most susceptibility alleles probably have only a small direct effect [6], instead acting to determine future disease risk mainly by their interaction with environmental exposures [7]. This process may occur through environmental modulation of gene expression (without alteration of the underlying genomic sequence) in a process known as 'epigenetics' [8].

Numerous genes have been identified through genome wide association studies (GWAS) and candidate gene approaches that appear to be associated, either directly or indirectly, with the regulation of body weight [9]. It is likely that, through a process of natural selection, these genes have become more prevalent due to the evolutionary advantage that they offer by promoting energy storage to survive periods of food deprivation. Within our obesogenic environment, however, these genetic susceptibility traits are now associated with an increased risk of obesity and associated metabolic diseases, such as Type 2 diabetes.

The majority of genes identified in monogenic cases of obesity appear to be involved in the central regulation of energy intake. In this regard, the most strongly replicated candidate gene has been the melanocortin 4 receptor, which ha been suggested to be responsible for up to 6% of cases of severe, early onset obesity [10], but also likely contributes to population variations in body fat, adipose tissue distribution, some metabolic traits and childhood weight gain [11]. Genes involved with energy utilization have also been implicated in common obesity, with replicated associations shown for genes encoding β-adrenergic receptors 2 and 3, hormone-sensitive lipase, and mitochondrial uncoupling proteins 1, 2, and 3 [12]. The FTO gene is another example of a key gene that appears to be responsible for population-wide variations in body weight and composition [13], and represents just one of many genes identified in recent times [14]. This is a rapidly progressing field of research, in terms of both the identification of new genes and the role that they play in both adult and early onset weight gain [15-17].

The role of epigenetics in body weight regulation is less clear, however, and remains the focus of intense interest [18]. Epigenetic factors include several different classes of modified nucleotides and proteins that interact to regulate the activity state of underlying DNA sequence. Such factors are usually heritable throughout cell division and play a pivotal role in specifying cell fate and function. The methylation of specific CpG dinucleotides (the most widely studied epigenetic mark) is known to affect gene expression and to be sensitive to environmental disruption, including dietary change [19-23]. Epigenetic profile is modifiable during critical developmental periods [23-26] and is involved in several obesity-related metabolic pathways [26,27]. Epigenetic analyses, including DNA methylation profiling, thus offers a compelling new paradigm for how early nutrition may impact upon later obesity [28] and, although studies assessing the role of DNA methylation in human obesity are rare [29,30], the challenge is to now develop specific studies aimed at investigating how environmental exposures interact with underlying genetic determinants to dysregulate gene expression and lead to metabolic disorders [31].

While societal change could theoretically solve the problem of childhood obesity, current endeavours have not proven fruitful in the long-term [32] and millions remain at high risk of developing weight-related disease over the next decade. Public health initiatives to combat childhood obesity must be complemented by effective identification and management programs for individuals at high risk of developing weight-related disease.

### Childhood obesity and its relationship with health-related complications

Interactions between obesity, genetics and the environment may be important not only to the development of obesity itself but also to its co-morbidities. For example while obese individuals are more likely to develop cardiovascular disease, once the disease is established then obesity may actually offer some protection against its adverse outcomes [33] - a paradox coined 'reverse epidemiology'. Furthermore, investigation of weight-related co-morbidities in adult populations has revealed a subpopulation of metabolically 'healthy' obese individuals [34]. It is not yet clear whether such individuals will continue to remain disease-free, or whether this phenotype represents delayed disease onset.

Regardless, this phenotype is relevant to how we approach the obesity epidemic in youth: specifically, some children with excess adiposity will remain disease-free while others will go on to have metabolic disease in their teenage years or in young adulthood. As the demand for intensive weight management treatment vastly exceeds the available supply, some method of efficaciously allocating limited resources must be developed. Additionally, identifying factors that predict weight-related co-morbidities may lead to strategies to prevent such complications at the population or primary care level, thus giving patients and practitioners more time to resolve the underlying weight problems.

Understanding the complex interactions involved in the development of weight-related disease necessitates a holistic approach to research, rather than the more traditional 'reductionist' mode. In a 'systems biology' approach, investigations are used to systematically study complex interactions in biological systems [35]. Until very recently, this has been impractical for the study of human obesity but such endeavours are now becoming possible through major technical and computational advances. Furthermore, there has been an increasing recognition within the scientific community that it is important to gather complementary data across cohorts, both to increase sample size and to avoid bias for population generalization [36,37]). Together, these approaches offer a unique opportunity to the study of complex 'gene × environment' (G×E) interactions. 'Nutrigenomics' (the field of research assessing the effect of nutrition on gene expression) is one important example [38].

To date, most clinical research has centred around *either *identifying genetic susceptibility in specific groups of obese children [39] or studying the impact of specific environmental factors on weight gain [40]. To fully explore the heterogeneous factors associated with weight-related disease, however, studies should combine comprehensive phenotypic description (ideally incorporating clinical physical examination), with the capacity to examine genetic/metabolic/proteomic characteristics and accurate measures of environmental exposure. To disentangle associations from potentially causative factors, such studies would recruit initially disease-free overweight/obese youth for standardised clinical longitudinal follow-up to identify important clinical endpoints as they develop.

### What can a Biorepository offer for the study of childhood obesity?

A biorepository acts as a library of biological and associated data from large numbers of individuals, either from the general population or from specific groups of individuals with a condition of interest. Moving beyond isolated sample collection, biorepositories now comprise comprehensive and sophisticated data warehouses containing biological material, results from associated biochemical investigations, detailed participant-level information (e.g. self-completed questionnaires and clinical records) and area-level variables (e.g. measures of environmental exposures).

Such initiatives should, in due course, provide the necessary statistical power to detect relatively small direct effects (odds ratios of the order of 1.15 to 1.3) that will nonetheless be of clinical significance and be directly relevant to public health [41]. There are now many established biobanks, with examples being the Western Australia Genome Project (http://www.genepi.org.au/projects/waghp.html) and the UK Biobank (http://www.ukbiobank.ac.uk). Each aims to recruit enough individuals for stand-alone genetic association studies, although future international harmonisation of national biobanks (e.g. through ventures such as the Promoting Harmonisation of Epidemiological Biobanks in Europe (PHOEBE); http://www.populationbiobanks.org) will further increase statistical power [41].

The investigation of complex childhood weight issues should similarly benefit by routinely and prospectively 'biobanking' blood samples (and possibly other biofluids such as urine and saliva) alongside longitudinal data from sufficient numbers of overweight and obese youth. No such biobank has yet been established. This manuscript describes the initial phase of establishing the 'Childhood Overweight BioRepository of Australia' (termed 'COBRA'). The aim in publishing protocols at this early stage is to enable harmonisation and collaboration with other centres who may be considering such investigations. It is hypothesised that, given sufficiently harmonised data and a multidisciplinary, international, multi-site approach, COBRA will ultimately enable systems biology investigations of the effects of excess adiposity from childhood across multiple biological pathways, including the G×E interactions that impact on these molecular networks.

### Which specific questions will be addressed within COBRA?

Initial investigations will consist of cross-sectional analyses of prevalent cases (comparing those with and without co-morbidities at initial assessment). Longitudinal data collection will enable investigations of incident cases (those who subsequently develop co-morbidities) and those who remain disease free (controls). Specific objectives of COBRA are outlined below:

#### 1. Identification of factors associated with increased risk of weight-related co-morbidity at initial presentation

There is a high prevalence of adverse health problems within cohorts of overweight children. For example, abnormal glucose metabolism (i.e. either impaired fasting glucose or impaired glucose tolerance) is present in approximately 10% of obese youth, whereas the metabolic syndrome is present in as many as 1-in-4 obese individuals attending specialist services [42]. While discrepancies in diagnostic criteria for the latter lead to some variation around this figure, a diagnosis of metabolic syndrome in pre-adult years is highly significant [43]. A biorepository designed to identify risk factors associated with weight-related complications, and the development and assessment of appropriate screening and treatment strategies to prevent or delay co-morbidity onset, would greatly assist clinical management.

#### 2. Determination of how environmental, genetic and metabolic factors come together to confer an increased risk of future disease

With prospective data collection, it will be possible to begin to discriminate temporal relationships between G×E 'clusters' and development of specific co-morbidities. These data should inform service delivery and allow appropriate management strategies to be focussed to those most at-risk. COBRA will build upon evidence from existing research strategies that are examining gene by nutrition interactions (http://www.nugo.org/) and physical activity by metabolism interactions (http://www.earlybirddiabetes.org/index.php) in healthy non-obese adults and children respectively, as well as gene by nutrition interactions in obese adults (http://www.nugenob.com/). The project carves an important niche in this regard.

#### 3. Identification of factors associated with success in long-term weight loss and maintenance

With prospective data collection, it will be possible to identify factors associated with success in controlling weight and avoidance of long-term disease. Within intensive weight management programs some children do well, while others appear extremely resistant to change [44-46]. Some of this resistance is likely to be due to complex individual family, lifestyle, societal and cultural factors, but there is also evidence that metabolic [47], genetic [48,49] and environmental factors [44] also impact upon potential 'success'. While the US National Weight Control Registry (http://www.nwcr.ws/) has yielded important and unexpected information for overweight adults, there is no comparable information for children. The extensive baseline phenotyping undertaken in COBRA offers such an opportunity for similar studies in children.

This manuscript outlines the initial phases of development of COBRA, with details of study design and preliminary data relating to uptake.

## Methods

### Participants: Who should be approached for inclusion?

COBRA was established to investigate the development of weight-related co-morbidity, rather than the development of obesity *per se*, and therefore it targets populations with established obesity. Sites that can support in-depth clinical assessment are best placed to join COBRA although the project was designed using a 'modular' format so that it is sufficiently flexible to cope with variations in the specific study components that can be implemented according to feasibility at the local site.

At the parent site (The Royal Children's Hospital (RCH), Melbourne, Australia), all overweight and obese patients referred to the Weight Management Service are approached for enrolment. For the purposes of recruitment, overweight and obesity is classified using the 85^th ^and 95^th ^percentile cut-offs of US-derived data produced by the Centres for Disease Control and Prevention (http://www.cdc.gov), as these data are routinely used on growth charts in Australia. When it comes to longitudinal analysis, there are a range of alternative systems for classification (e.g. International Obesity Task Force criteria, or World Health Organisation recommendations) and these may continue to evolve over time. Therefore, the COBRA dataset will always include raw height, weight and BMI data, so that other classifications can be used flexibly and as appropriate. All children and adolescents (up to age 17.99 years) are approached, with no minimum age for recruitment recognising that, realistically, very few (if any) children are referred and routinely managed through specialist weight management services before the age of 2 years.

With 200-250 new patients each year, this is the largest tertiary-hospital based service within the limited services offered across Australia [50]. The spectrum of care follows that of other tertiary hospital paediatric weight management services [46]. Routine clinical care involves three-monthly patient visits. In addition to patients with established co-morbidities at initial presentation, a significant proportion develop co-morbidities over time, such as insulin resistance, dyslipidaemia and hypertension. Some go on to develop established diseases such as type 2 diabetes and the metabolic syndrome [51] in adolescence/early adult life. These will act as cases to the 'healthy' overweight/obese controls within the setting of a prospective cohort study.

Recruitment is also being undertaken, with specific ethical approval, within a National Health and Medical Research Council (NHMRC)-funded Randomised Controlled Trial that is currently assessing a 'shared care' approach to the management of childhood obesity (Australian Clinical Trials Registry 12608000055303; http://www.rch.org.au/ccch/research.cfm?doc_id=12126). Two additional clinical centres and one population-based cohort study (that includes a significant clinical examination component) are also currently applying for site-specific ethical approval to join the COBRA project. While longitudinal data collection is not a prerequisite for COBRA uptake, it is emphasised to disentangle temporal associations of future disease.

### Measures: What data should COBRA collect?

A biorepository needs to balance comprehensive data collection against a patient's clinical needs as this could jeopardise either recruitment or long-term follow-up. Furthermore, data collection should not adversely impact upon, or drive, routine clinical treatment protocols. Measures should include comprehensive assessment of environmental exposure, alongside collection of biosamples that will allow a systems biology approach through genetic, epigenetic and metabolic testing. Accurate anthropometry is vital, as are detailed data on clinical management and follow-up.

Multi-centre data pooling and analysis requires universal agreement relating to key measures. Perhaps the biggest challenge is obtaining agreement on which measures of energy intake and expenditure to collect. Such harmonisation can be enhanced by communication across networks of experts, such as the 'Australasian Child and Adolescent Obesity Research Network' (ACAORN; http://www.acaorn.med.usyd.edu.au/) and the German competence network of obesity (Kompetenznetz Adipositas) of the German ministry of Research and Education (http://kn-adipositas.de/knadipositas/default.aspx).

Data collection components employed in COBRA can be summarised as those relating to environment, anthropometry and clinical evaluation, and biological samples, plus long-term follow-up clinical data. These are described in more detail below and in Table 1.

**Table 1:** Parameters collected for each participant within COBRA.

Self-completed participant information **Domain: Instrument/Source (respondent**^**1**^)	Clinical exam	Blood samples
• Health: Self-rated health (P, C) • Concern with weight: HopSCOTCH^2 ^(P) • Help seeking: HopSCOTCH^2 ^(P) • Pregnancy & birth: HopSCOTCH^2^, LSAC^3^, NHS^4 ^(P) • Early nutrition: LSAC^3 ^(P) • Family health history: HopSCOTCH^2 ^(P) • Mental health: **SDQ**^**5 **^(P, C); **Kessler 10**^**6 **^(P, C) • Enjoyment of physical activity (PA): LEAP^7 ^(P, C) • Active/Sedentary time: HopSCOTCH^2^, LSAC^3 ^(P, C) • Targeted nutrition/PA: HopSCOTCH^2 ^(P, C) • Transport, biking, walking: **IPAQ**^**8 **^(P, C) • Childcare: HopSCOTCH^2 ^(P) • Sleep habits: LSAC^3 ^(P, C) • Neighbourhood: LSAC^3^, ALSPAC^9^, **NEWSA**^**10 **^(P, C) • Socio-demographic: Census (P) • Household composition: FLAME^11 ^(P) • Nutrition: 3-day prospective food diary (P, C); **ACAES**^12 ^(P, C) • Quality of life (QoL): **PedsQL Core module**^13 ^(P, C); **Sizing Them Up**^14 ^(P); **Sizing Me U**p^15 ^(C) • **MARCA**^16^: 24-hour PA recall	Clinical history • Specific details relating to weight • Other health issues • Peri natal and Past Medical History • Medications (past and present) • Allergies • Immunisations • Developmental history and schooling • Family history • Sleep issues	Clinicalpathology • Oral Glucose Tolerance Test • Fasting glucose • Fasting Insulin • Lipid profile • Thyroid function test • Liver function tests • Iron studies • Full blood count • Haemoglobin A1C • Vitamin D • Vitamin B12 • Magnesium • Calcium • Phosphate • Folate
	Clinical examination • General appearance (dysmorphism, affect, body proportions) • Cardiovascular examination including blood pressure • Respiratory examination • Abdominal examination • Skin (acne, hirsutism, acanthosis nigricans, intertrigo, striae) • Pubertal assessment (method of Tanner and Whitehouse)	Research assays
	Anthropometry	See Table 2
*Notes: *In addition to self-completed survey items, participants wear an accelerometer, which is an omni-directional device to record child's movement in 15 second intervals over 9 days. О	• Height (measured to nearest • 0.1 cm using a stadiometer) • Weight • Waist circumference • Bioimpedence (measure of % fat by body quadrant, fat free mass, & basal metabolic rate)	

^1 ^P = parent, C = child (most instruments are completed by children approximately aged 11 years and older, exceptions are QoL instruments and accelerometry). Data sources indicated in **bold **are validated instruments.

^2 ^HopSCOTCH is an NHMRC-funded clinical intervention trial of shared care for paediatric obesity currently being undertaken at RCH/MCRI (http://www.rch.org.au/ccch/research.cfm?doc_id=12126)

^3 ^Longitudinal Study of Australian Children (http://www.aifs.gov.au/growingup/)

^4 ^National Health Survey 2004/05 (http://www.abs.gov.au/)

^5 ^Strengths & Difficulties Questionnaire (http://www.sdqinfo.com/).)

^6 ^Kessler 10 [66].

^7 ^Live Eat and Play Study [67]

^8 ^International Physical Activity Questionnaire (http://www.ipaq.ki.se/ipaq.htm)

^9 ^Avon Longitudinal Study of Parents and Children (Children of the 90's; http://www.bristol.ac.uk/alspac/).

^10 ^Neighborhood Environment Walkability Scale Abbreviated (sections C-H; http://www.activelivingresearch.org/node/10649): only administered to 13+ yo

^11 ^FLAME--A New Zealand study to identify risk factors for obesity in children [68]

^12 ^The Australian Child & Adolescent Eating Survey food frequency questionnaire (ACAES FFQ), [69]

^13 ^PedsQL - Measurement model for the Pediatric Quality of Life Inventory (http://www.pedsql.org/).

^14 ^'Sizing Them Up' [70]

^15 ^'Sizing Me up' [71]

^16 ^Multimedia Activity Recall for Children & Adolescents (MARCA) [72]: this instrument is currently waiting to be implemented into the study protocol.

a) Environmental measures collected within COBRA are primarily collected through self-completed questionnaires. Wherever possible, these have been validated and/or are standardised and widely used. COBRA participants complete questionnaires at home in the week preceding the initial clinical appointment. These include a parent questionnaire, a child questionnaire (for those aged 11 years and older), a food frequency questionnaire, and a 3-day food diary. Parent and child questionnaires are available as supplementary material to this manuscript (Additional files 1, 2, 3, 4, 5, 6, 7, 8, 9, 10, 11, 12, 13, 14). Children aged five years and older also complete a specific child survey upon clinical presentation (interviewer-administered for the youngest children). At this time, parents also complete the parent-reported version of these instruments so that items and reference periods are synchronised between parents and child.

b) Biological samples are collected for both clinical and research applications. RCH Weight Management Service clinical protocols include a routine panel of pathology examinations that is subject to minor modification based on individual clinical presentation. It includes: (i) metabolic profiles (including liver function tests, lipid profiles, insulin and glucose [fasting in under 10 year olds and as part of an oral glucose tolerance test in over 10 year olds]), and (ii) nutritional assessment (iron stores, B group vitamins, Vitamin D, folate, calcium, phosphate, magnesium).

Research specimens are collected at the same time as clinical pathology samples to minimise patient burden and distress. Table 2 outlines the protocol for research biological sample collection and storage. These samples are then biobanked for future investigation which will include: (i) metabolic analyses (e.g. adiponectin, leptin, resistin), (ii) genetic analysis, (iii) gene expression analysis, and (iv) epigenetic analysis in peripheral blood mononuclear cells. Genetic analyses will include major susceptibility loci for weight-related co-morbidities such as T2DM [52], lipid abnormalities [53] and the metabolic syndrome [54], as well as larger-scale genome-wide association studies. Expression profiling, by high throughput quantitative RT-PCR, will include assessment of mRNA expression of known target genes involved in obesity, type 2 diabetes, metabolism and inflammation, while DNA microarray analysis may potentially reveal novel gene pathways significantly altered in overweight children at highest risk of metabolic complications. Epigenetic analysis will include assessment of DNA methylation of CpG dinucleotides involved in the regulation of gene expression, as this is strongly influenced by environmental factors. Biobanked samples are currently stored within the Murdoch Childrens Research Institute Biobanking Facility, which will ultimately be housed in the Children's BioResource Centre in the new Royal Children's Hospital in Melbourne. As COBRA moves to multi-site data collection, local sites will need to assess the feasibility of maintaining biospecimen locally or centralising sample storage using existing COBRA infrastructure.

**Table 2:** COBRA Biobank sample collection.

		Biospecimen fractions & number/volume of stored aliquots					
Participant age	Type of collection tube	Guthrie cards	Peripheral whole blood	Plasma	Lymph-ocytes	Granulo-cytes	Sera
> = 3 years	EDTA	4 × 0.1 ml		2 × 1 ml	3 × 1 ml	2 × 1 ml	
	SG						6 × 0.5 ml
	LH		4 × 1 ml	4 × 0.5 ml			
	Citrate			4 × 0.25 ml			
< 3 years	EDTA	4 × 0.1 ml			2 × 1 ml	2 × 1 ml	
	SG						4 × 0.5 ml
	LH		2 × 1 ml	2 × 0.5 ml			
	Citrate			3 × 0.25 ml			

Number of samples × volume collection. ml = millilitre(s). EDTA = EthyleneDiamineTetraAcetic acid tube. SG = Serum Gel tube. LH = Lithium Heparin tube.

c) Anthropometry and clinical data collection includes baseline and at least bi-annual measures of height, weight, waist circumference, total and regional fat and muscle mass (using a body composition analyser (currently BC-418, Tanita Corporation, Tokyo, Japan)), blood pressure and pubertal status. Additionally, the initial consultation collects detailed information relating to the presence of weight-related co-morbidities.

d) Longitudinal follow-up data are collected through clinic re-attendance up until patients reach 18 years of age. These clinic visits are scheduled at equally spaced intervals (initially 3 monthly), with routine clinical data collected (including Auxology, all details relating to attempts at weight management, other clinical events, and results of routine repeat clinical investigations). Follow-up in adulthood for the parent organisation will be enabled through participation in BioGrid (http://www.biogrid.org.au/wps/portal), a Victorian data-linkage resource that can identify and facilitate access to data on medical visits at other participating hospitals or health administration datasets maintained by the Victorian Department of Health. As such data linkage resources may not be available for other national or international centres, and recognising that some children will fail to reattend follow-up appointments, COBRA has also incorporated a 'consent to re-contact' procedure should participants be lost to follow up through standard clinical contact.

**Figure 1:**
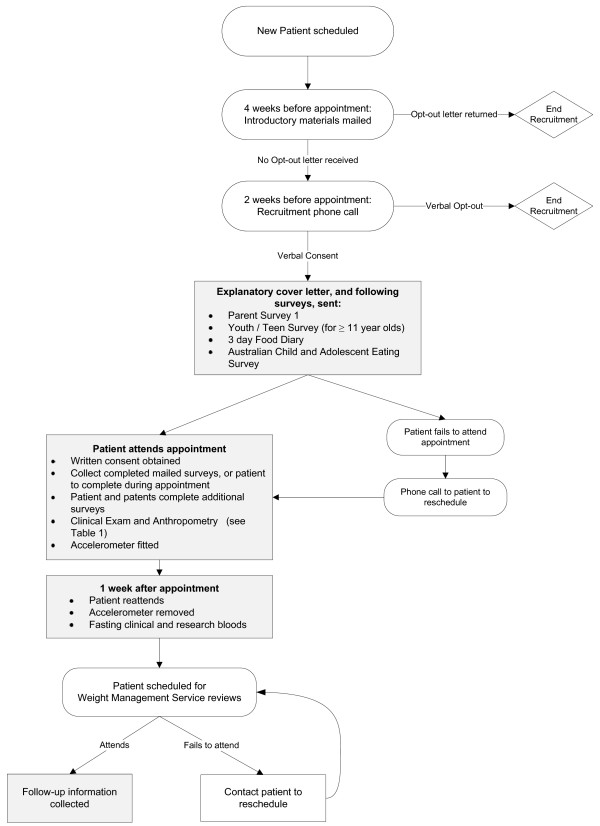
**COBRA Recruitment and Baseline Processes**.

The process of recruitment, along with data and sample collection, is shown as a flowchart in Figure 1.

It is intended that data will be stored centrally (subject to local patient confidentiality constraints) using the Western Australian Genetic Epidemiology Resource (an NHMRC Enabling Facility; http://www.wager.org.au/) or similar resource. This facility provides rigorous patient privacy and confidentiality safeguards, especially in relation to the transmission of electronic information, combined with the ability to customise access to specific data components (e.g. individual variables) and/or patient data (e.g. subsets of participants).

### What is the anticipated attrition?

Obesity management is associated with notoriously poor compliance [55]. Therefore steps that improve rates of follow-up and clinic attendance will improve the quality of longitudinal data. In a recent audit of the RCH Weight Management Service, non-attendance was 20% (cancellations 7%, failure to attend without prior notice 13%) over a 3 ½ year period. This high rate of patient treatment attendance is predominantly due to the addition of a clinic co-ordinator who contacts patients and ensures that all clinic appointment times are filled. While these figures appear favourable for the parent organisation, there may be large inter-site variations in clinic compliance.

### Ethical issues

COBRA, or any comparable childhood biorepository, involves complex ethical issues. These include data collection from minors, long-term storage of biological samples for subsequent genetic analysis, participant expectations, data protection and privacy, data linkage, and unspecified consent. These issues have been extensively reported elsewhere [56-59]. Despite these complex challenges, there is widespread support for projects that will benefit the public good [60]. COBRA has been approved at the parent site by the RCH Human Research Ethics Committee and current COBRA investigators can provide support, in terms of access to approved ethical documents and consent forms, to other investigators seeking to join this initiative. Fully informed and written consent is obtained in all cases.

### Sample size and statistical power

Studies of G×E interactions require large sample sizes. At a population level, the study of G×E interactions in disease development requires tens of thousands of subjects and is driven by the low frequency of disease within the population (e.g. the UK Biobank - http://www.ukbiobank.ac.uk/). Conversely, however, much smaller numbers are required when the prevalence of the condition of interest is much higher within the study population. Therefore, biobanking samples from groups of individuals who are at much higher risk of disease (e.g. overweight youth or those with a family history of disease) is likely to be as productive as population-based biobanks, in terms of the number of cases identified, at a much smaller total sample size [61].

Data on the specific contribution of genetic, environmental, and G×E interactions to the development of co-morbidities in obese youth is not currently available - this is one reason for initiating COBRA. Sample size estimates for COBRA were therefore developed based on hypothetical combinations of at-risk allele frequencies, environmental exposure prevalence and magnitude of G×E interactions. The sample size estimates are applicable to logistic regression models (to estimate the size of G×E interaction on dichotomous endpoints defined by disease status), and Cox's proportional hazards models (to analyse time until disease occurrence estimating the increase in the instantaneous rate of disease) resulting from the G×E interaction.

Table 3 presents the predicted power under several scenarios. This indicates that with 1000 children, COBRA will have 80% power to detect G×E interactions at the 5% level of significance in half of the modelled combinations. This improves substantially with a sample size approaching 2000 subjects. Given current national and international uptake of COBRA, it is anticipated that this will be achievable within 3-5 years.

**Table 3:** Power to detect Gene × Environment (G×E) interactions.

	SAMPLE SIZE = 1000			SAMPLE SIZE = 2000		
	G×E interaction OR = 2			G × E interaction OR = 2		
Allele frequency (%)	Binary environmental exposure prevalence (%)			Binary environmental exposure prevalence (%)		
	20	30	40	20	30	40
10	37.6%	45.8%	49.5%	64.2%	74.6%	78.6%
20	49.8%	58.9%	62.2%	79.0%	**87.1%**	**89.4%**
30	50.4%	58.7%	61.2%	79.6%	**87.0%**	**88.7%**
	**G × E interaction OR = 2.5**			**G × E interaction OR = 2.5**		
Allele frequency (%)	Binary environmental exposure prevalence			Binary environmental exposure prevalence		

	20	30	40	20	30	40
10	57.2%	67.9%	72.3%	**85.7%**	**92.9%**	**95.0%**
20	72.6%	**81.7%**	**84.3%**	**95.2%**	**98.2%**	**98.7%**
30	73.3%	**81.3%**	**83.1%**	**95.5%**	**98.1%**	**98.5%**
	**G × E interaction OR = 3**			**G × E interaction OR = 3**		
Allele frequency (%)	Binary environmental exposure prevalence			Binary environmental exposure prevalence		

	20	30	40	20	30	40
10	71.4%	**81.9%**	**85.7%**	**94.7%**	**98.2%**	**99.0%**
20	**85.9%**	**92.5%**	**94.1%**	**99.0%**	**99.8%**	**99.9%**
30	**86.6%**	**92.2%**	**93.1%**	**99.1%**	**99.8%**	**99.8%**

OR = odds ratio. Disease prevalence is 25% throughout. Magnitude of associations between binary outcome and each of the gene and environmental risk factors are 1.6 throughout. Condition is assumed to be dominant throughout. Figures in bold represent scenarios where sufficient power for the study will be achieved.

The proportion of participants with specific adverse health problems will be reported with 95% confidence intervals. Allele frequencies and presence of environmental factors of interest will also be summarised. Logistic regression models and Cox proportional hazards models for binary and time to event outcomes respectively, will be fitted to identify the risk and protective factors (predictor variables) for health problems and long-term disease (outcomes) for obese children.

Logistic and Cox regression models will be fitted to test the hypothesis that the effect of specific alleles on the risk of developing adverse health problems and long-term diseases is modified by the presence of select environmental factors (i.e. is the size of the odds ratio (or hazard ratio) between a particular gene and a specific health problem dependent upon whether the environmental risk factor is present). In these models the gene and environment variables (both categorical) will be used in the model as predictor variables in addition to a variable that represents the interaction between the gene and environment variables. The p-value for the gene-by-environment interaction variable will be used to quantify evidence against the hypothesis that there is no effect modification (interaction).

Where the effects of the gene on the disease are not in opposing directions within the categories of the environmental exposure, evidence for a gene-disease effect will be quantified using a simultaneous (or "joint") hypothesis test

[62] of the gene and gene-by-environment interaction effects. There is recent evidence that this joint test, as opposed to a test of the gene variable alone in a simple marginal gene-disease model, improves power for detecting a gene effect - even in the presence of a degree of misclassification in the environmental measures [62]. The results from the interaction tests, supplemented by the joint tests where appropriate, will clarify the nature of the specific gene-disease associations.

## Results

Data collection for COBRA at the RCH Weight Management Service was initiated in late April 2009. In the first nine months, 116 patients were approached for study participation. Of these, 24% cancelled or repeatedly failed to attend their appointment and were subsequently excluded. Of the 87 remaining patients, 25% declined participation, 67% consented to COBRA, and 8% are currently being recruited.

Table 4 presents demographic information and response rates for the first ten months of recruitment. Of the 77 eligible families that have attended their initial Weight Management Service appointment, early findings indicate lower participation rates among adolescents.

**Table 4:** COBRA response rates by age and gender.

		Sex		Age ranges (years)		
	**Total number**	**M**	**F**	**<5**	**5-10**	**11-18**

**Eligible (%)**	77	41 (53)	36 (47)	6 (8)	34 (44)	37 (48)

**Enrolled (%)**	55	30 (55)	25 (45)	6 (11)	25 (45)	24 (44)

**Declined (%)**	22	11 (50)	11 (50)	0 (0)	9 (41)	13 (59)

Participants with upcoming initial appointments (n = 3) and families currently being recruited (n = 7) have been excluded from these results.

Completion rates for the various individual components of baseline data collection are as follows: (a) Parent Survey 1, 85%; (b) Child Survey 1, 91%; (c) Parent Survey 2, 85%; (d) Child Survey 2, 97%; (e) food frequency questionnaire, 93%, (f) 3-day food diary, 56%; (g) accelerometry, 92%; and, (h) venous blood sample, 73% (with an additional 11% scheduled). This indicates that, despite reports in the literature indicating high rates of treatment non-adherence, families that participate in COBRA demonstrate a high rate of baseline data completion.

Some follow-up visits have already occurred, but data relating to these have not yet been analysed.

## Discussion

The establishment of a biorepository of data and biological samples from large numbers of overweight and obese children, alongside longitudinal data collection relating to co-morbidities, offers a unique opportunity to examine the complex G×E interactions that likely govern long-term disease risk in children and adolescents.

One of the primary strengths of COBRA is that it incorporates comprehensive baseline clinical phenotyping with longitudinal follow up. Future investigations may be incorporated through (a) on-going clinical follow-up by established tertiary treatment services, or (b) consent to recontact for future waves of data collection. Multi-site input, both nationally and internationally, will allow specific investigation of G×E effects in diverse populations, as well as allowing analysis of comparative effectiveness of different site-specific treatment modalities. Additionally, combining research biospecimen collection (for storage in the biorepository) with pathology examinations required for clinical care has resulted in (a) access to specialist phlebotomists available through the tertiary paediatric care sector, (b) reduced patient distress, and (c) a high proportion of patients successfully contributing research samples. Routine clinical follow up has also resulted in on-going contact with families and multiple opportunities to collect and query self-completed questionnaire responses, as indicated by high response rates for this component.

The primary potential weakness of COBRA is low recognition of childhood obesity by parents [63] and health professionals [64] as well as a paucity of well-resourced services for referred children [50]. Furthermore, as COBRA recruitment occurs within specialist centres, selection bias from recruitment of only treatment-seeking individuals will occur. There may also be variation in allele frequencies between sites due to differences in the underlying population substructure. While this may increase samples sizes required for the analysis of G×E interactions, it may also provide information on potential confounding due to ancestry and facilitate identification of different G×E interactions across different sites/countries.

## Conclusions

To date, current strategies aimed at both the prevention and treatment of childhood obesity, and especially within primary care settings, have largely failed to deliver expected results [65]. This could lead to a pandemic of weight-related disease unless novel modes of research are urgently developed to prevent and treat those most at risk. Recent technological advances now allow a 'wider' approach to the study of factors that govern weight status and disease risk in young people. A biorepository of data and biological samples from a large cohort of obese children and adolescents will become a valuable resource over time, enabling investigation of paediatric precursors to adult disease.

Many may be of the opinion that the problem of childhood obesity does not require this intense level of investigation. Some argue that the problem can be easily solved with more prescriptive measures to modify lifestyles of young children. All attempts at this to date, however, have inexorably failed in the long-term. It is therefore time to rethink strategy, with biorepository-based initiatives likely to prove an invaluable resource in years to come.

## Declaration of competing interests

The authors declare that they have no competing interests.

## Authors' contributions

MS conceived and designed the study, led development of study protocols, assisted in obtaining ethical approval, and is actively involved in recruitment. SC has assisted in development of protocols, led procedures to obtain ethics approval, and is responsible for the day-to-day running of the study. RS, MC and GW assisted in study design and protocol development, with RS taking a leading role in the procedures required for biobanking of samples. ZM is actively involved in recruitment. WK, NC, JW, GL have been involved in the national and international development of study design and protocols. OU provided statistical input to the study design. MW assisted in study design and protocol development. MS, MW and RS have co-led the development of the study from conception to current practice. All authors read and approved the final manuscript.

## Pre-publication history

The pre-publication history for this paper can be accessed here:

http://www.biomedcentral.com/1471-2288/10/100/prepub

## Supplementary Material

Additional file 1 **A. COBRA Survey 1 Parent 1-2yo.pdf**. COBRA Survey 1 for parents of 1-2 year olds Click here for file

Additional file 2 **B. COBRA Survey 1 Parent 3-4yo.pdf**. COBRA Survey 1 for parents of 3-4 year olds Click here for file

Additional file 3 **C. COBRA Survey 1 Parent 5-10yo.pdf**. COBRA Survey 1 for parents of 5-10 year olds Click here for file

Additional file 4 **D. COBRA Survey 1 Parent 11yo.pdf**. COBRA Survey 1 for parents of youth aged 11 years and older Click here for file

Additional file 5 **E. COBRA Survey 1 Youth 11-13yo.pdf**. COBRA Survey 1 for youth aged 11-13 years Click here for file

Additional file 6 **F. COBRA Survey 1 Teens 14yo.pdf**. COBRA Survey 1 for teens aged 14 years and older Click here for file

Additional file 7 **G. COBRA Survey 2 Parent 1yo.pdf**. COBRA Survey 2 for parents of 1 year olds Click here for file

Additional file 8 **H. COBRA Survey 2 Parent 2-4yo.pdf**. COBRA Survey 2 for parents of 2-4 year olds Click here for file

Additional file 9 **I. COBRA Survey 2 Parent 5-7yo.pdf**. COBRA Survey 2 for parents of 5-7 year olds Click here for file

Additional file 10 **J. COBRA Survey 2 Parent 8-12yo.pdf**. COBRA Survey 1 for parents of 8-12 year olds Click here for file

Additional file 11 **K. COBRA Survey 2 Parent 13yo.pdf**. COBRA Survey 1 for parents of youth aged 13 years and older Click here for file

Additional file 12 **L. COBRA Survey 2 Child 5-7yo.pdf**. COBRA Survey 2 for children aged 5-7 years Click here for file

Additional file 13 **M. COBRA Survey 2 Youth 8-12yo.pdf**. COBRA Survey 2 for youth aged 8-12 years Click here for file

Additional file 14 **N. COBRA Survey 2 Teens 13yo.pdf**. COBRA Survey 2 for teens aged 13 years and older Click here for file
