# Supplementary material for: New directions in childhood obesity research: how a comprehensive biorepository will allow better prediction of outcomes
Source: BMC Med Res Methodol. 2010 Oct 22;10:100. doi: 10.1186/1471-2288-10-100 (PMC2984501; doi:10.1186/1471-2288-10-100)
Supplement: Additional file 1 — A. COBRA Survey 1 Parent 1-2yo.pdf. COBRA Survey 1 for parents of 1-2 year olds [file 1471-2288-10-100-S1.PDF]

1397163886

Participant code: | | | | | | | | | | | | | |

COBRA
Childhood Overweight
BioRepository of Australia

Survey 1 (Parent)

This survey is for the parents of children age 1 to 2 years old

Please bring the completed survey with you when you come to your next visit at The Royal Children's Hospital Weight Management Service.

You will give it to the researcher who meets you at this visit.

Thank you for agreeing to be in this study. This study is looking at things that may affect health problems for some overweight or obese children.

Before your child's first visit, we would like to get an idea about your child's general health, your pregnancy with this child, your family's health history, and some idea about your child's activities and neighbourhood. Please answer the questions on the next pages.

This should take you about 35-40 minutes.

It is private, and your answers are confidential.

INSTRUCTIONS

1. In this questionnaire, 'this/your child' means the child visiting the RCH Weight Management Service and who is enrolled in this study.
2. Please answer by filling in the circles completely like this ●
3. If you make a mistake, put a cross through it, then fill in and draw a circle around the correct one.
4. Use a blue or black pen only.
5. There are no right or wrong answers. If you aren't sure, just give the best answer you can. You can also make a comment in the margin - it will be read!
6. Please remember to fill in the back of each page as well.

Questions? Ring us (03) 9936 6512 or email us (mpowr@mcri.edu.au) any time

Thank you for taking part in COBRA

OFFICE USE ONLY

Date returned: ☐☐ / ☐☐ / ☐☐☐☐

Was survey completed before seeing clinicans? O No O Yes

Completed at: O RCH O Home O Other ____________________

Murdoch Childrens Research Institute — Healthier Kids. Healthier Future.
The Royal Children's Hospital Melbourne

A. General health

This study is looking at different things that may lead to health problems in overweight children. These things may be in the environment (for example, the foods that someone eats) or genetic (for example, due to genes in your DNA).

Some questions ask about the child's **biological** relatives. **Biological** relatives have genes and DNA in common because they are related in some way (e.g. the mother that gave birth to the child would be biologically related but a step-mother or step-father would not be).

For other questions, we are interested in the environment and it may not matter whether family members are biologically related (for example, family eating habits). We have tried to be clear when questions are about the biological relatives. Please ask if you have any questions or write any information you think would help us understand about your own family next to the question(s).

A.1. Who is completeing this form? (fill in one circle only)

- ○ Biological mother
- ○ Biological father
- ○ Other, please specify ______________________

A.2. Today's date: ☐☐ / ☐☐ / ☐☐☐☐
day month year

A.3. Child's date of birth: ☐☐ / ☐☐ / ☐☐☐☐
day month year

A.4. Child's gender: ○ Male ○ Female

A.5. In general, would you say your child's health is (fill in one circle only)

○ Excellent ○ Very good ○ Good ○ Fair ○ Poor

A.6. In the past SIX months, have you received any help or advice for any health concerns about your child?

- ○ No (go to question A.8)
- ○ Yes (go to question A.7)

A.7. Where did you get your help/advice from? (fill in one circle for each option)

No	Yes		No	Yes	
○	○	Other family members	○	○	Paediatrician
○	○	Friends	○	○	Other specialist doctor
○	○	GP (family doctor)	○	○	Parent group
○	○	Dietician	○	○	Books / written information
○	○	Maternal and Child Health Nurse	○	○	Web information
○	○	School nurse	○	○	TV program
○	○	Weight management service / information (e.g. Weight Watchers), please specify: ______________________	○	○	Other, please specify: ______________________

A.8. How concerned are you about your child's current weight? (fill in one circle only)

○ Not at all ○ A little ○ Moderately ○ Very

A.9. Have you sought any assistance to help manage your child's weight?

○ Yes ○ No

If **YES**, can you say from whom? (fill in all circles all that apply)

○ GP (family doctor)

○ Dietician

○ Paediatrician

○ Other (please specify) ____________________

A.10 Who is your child's usual doctor? (We will NOT contact your doctor)

Name of doctor ____________________

Name of practice ____________________ Postcode ☐☐☐☐

B. Your pregnancy and child's birth

B.1 Was the pregnancy for this child (fill in one circle only)

○ Planned, natural conception (go to question B.3)

○ Planned, assisted reproduction (go to question B.2)

○ Unplanned (go to question B.3)

B.2 If this pregnancy was planned (assisted reproduction), did it include (fill in one circle only)?

○ In vitro fertilization

○ Other, please specify ____________________

B.3 How old was the mother when she was pregnant with the child visiting the Weight Management Service?

☐☐ years (write 'unk' if unknown)

B.4 Approximately what was the mother's weight in the months right before she became pregnant with the child visiting the Weight Management Service?

☐☐☐.☐ kilos (write 'unk' if unknown)

B.5 About how much weight did the mother gain when she was pregnant with this child?

☐☐☐.☐ kilos (write 'unk' if unknown)

B.6. Did the mother ever get back to her pre-pregnant weight after she had this child (fill in one circle only)?

○ Yes, completely ○ Yes, nearly ○ No, heavier ○ No, lighter ○ Unknown ○ Doesn't apply

B.7 During the mother's pregnancy with this child, did she have (fill in one circle on each row)

No	Yes	Unknown	
○	○	○	Gestational diabetes?
○	○	○	High blood pressure?
○	○	○	Prescribed medicine/s? Please list: ________ ________
○	○	○	Other important illness(es)? Please list: ________ ________

B.8 What type of birth, or delivery, did your child have?

○ Normal ○ Breech ○ Caesarean ○ Vacuum extraction ○ Forceps ○ Other ○ Don't know

B.9 After how many weeks of pregnancy was your child born? [ ][ ] weeks

B.10 How much did your child weigh at birth? [ ][ ][ ][ ] grams **OR** [ ][ ] pounds [ ][ ] ounces

B.11 What was your child's length at birth? [ ][ ].[ ] cm (write 'unk' if unknown)

B.12 Was your child in a Neonatal Intensive care Unit or Special Care Nursery after he/she was born?

○ Yes (go to question B.13)

○ No (go to question B.14)

○ Don't know (go to question B.14)

B.13 Did he/she need breathing help from a ventilator machine after he/she was born?

○ Yes ○ No ○ Don't know

B.14 How old was your baby when you took him/her home from the hospital?

[ ][ ] days **OR** [ ][ ] weeks

B.15 During the pregnancy with this child, did the mother smoke at least one cigarette?

○ Yes (go to question B.16)

○ No (go to question B.17)

○ Don't know (go to question B.17)

B.16 On average, how many cigarettes did the mother smoke per day **during this pregnancy**? Please answer for each trimester of your pregnancy.

a) **First 3 months** of your pregnancy with this child	b) **Middle 3 months** of your pregnancy with this child	c) **Last 3 months** of your pregnancy with this child
○ None	○ None	○ None
○ 1 to 5	○ 1 to 5	○ 1 to 5
○ 6 to 10	○ 6 to 10	○ 6 to 10
○ 11 to 20	○ 11 to 20	○ 11 to 20
○ 21 to 30	○ 21 to 30	○ 21 to 30
○ 31 to 40	○ 31 to 40	○ 31 to 40
○ 41 to 50	○ 41 to 50	○ 41 to 50
○ 51 or more	○ 51 or more	○ 51 or more
○ Occasional - not everyday	○ Occasional - not everyday	○ Occasional - not everyday
○ Occasional - not every week	○ Occasional - not every week	○ Occasional - not every week

B.17 During the mother's pregnancy, did she, or anyone else, usually smoke inside the house?

○ Yes ○ No ○ Don't know

B.18 **During the pregnancy with this child,** did the mother ever have a full serve of alcohol (e.g. a full serve (standard drink) equals a 100 ml glass of wine, a nip of spirits, a glass/pot of regular strength beer, etc.)?

○ Yes (go to question B.19 & B.20)

○ No (go to section C)

○ Don't know (go to section C)

B.19 On average, **when the mother was pregnant with this child**, how many days per week did she have an alcoholic drink?

a) **First 3 months** of your pregnancy with this child	b) **Middle 3 months** of your pregnancy with this child	c) **Last 3 months** of your pregnancy with this child
○ None	○ None	○ None
○ 1	○ 1	○ 1
○ 2	○ 2	○ 2
○ 3	○ 3	○ 3
○ 4	○ 4	○ 4
○ 5	○ 5	○ 5
○ 6	○ 6	○ 6
○ 7	○ 7	○ 7
○ 2-3 days a month	○ 2-3 days a month	○ 2-3 days a month
○ About 1 day a month	○ About 1 day a month	○ About 1 day a month
○ Less often	○ Less often	○ Less often

B.20 On average, when the mother was pregnant with this child, on days that the mother would have a standard drink, about how many standard drinks did she usually have? (A standard drink equals a 100ml glass of wine, a nip of spirits, a glass/pot of regular strength beer, etc.)

a) **First 3 months** of your pregnancy with this child	b) **Middle 3 months** of your pregnancy with this child	c) **Last 3 months** of your pregnancy with this child
○ None	○ None	○ None
○ 1 or 2	○ 1 or 2	○ 1 or 2
○ 3 or 4	○ 3 or 4	○ 3 or 4
○ 5 or 6	○ 5 or 6	○ 5 or 6
○ 7 or 10	○ 7 or 10	○ 7 or 10
○ 11 to 12	○ 11 to 12	○ 11 to 12
○ 13 or more	○ 13 or more	○ 13 or more

C. Your child's early eating habits

Now, we are going to ask some questions about when your child was a baby and about his/her early food experiences.

We will ask several questions about how old your child was when they had some types of foods, like formula, milk, or solid food. We understand that families may have started these types of foods at very different times. This is why we have given you the choice of entering the time in days, weeks, months or years.

For each question, please write in the **number** of days, weeks, months or years.

C.1 Was this child ever breastfed? (including colostrum in first few days after birth)

○ Yes (go to C.2) ○ No (go to C.3) ○ Don't know (go to C.3)

C.2 How old was this child when he/she completely stopped being breastfed (including expressed breast milk)?
Please enter the number of days, weeks, months or years (choose one) or fill in the circle of the correct response

[ ][ ][ ] days **OR** [ ][ ][ ] weeks **OR** [ ][ ] months **OR** [ ][ ] years **AND** [ ][ ] months

○ Other, please explain: ____________________

○ Don't know

C.3 How old was this child when he/she was first given infant formula regularly (regularly means more than twice a week for several weeks continuously)?
Please enter the number of days, weeks, months or years (choose one) or fill in the circle of the correct response

[ ][ ][ ] days **OR** [ ][ ][ ] weeks **OR** [ ][ ] months **OR** [ ][ ] years **AND** [ ][ ] months

○ Never given formula regularly

○ Other, please explain: ____________________

○ Don't know

C.4 How old was this child when he/she first had any regular milk (e.g. cow's milk, soy milk) other than formula or breast milk (include colostrum as breast milk)?
Please enter the number of days, weeks, months or years (choose one) or fill in the circle of the correct response.

[ ] days **OR** [ ] weeks **OR** [ ] months **OR** [ ] years **AND** [ ] months

- ○ Other, please explain: ______
- ○ Don't know

C.5 How old was this child when he/she first had **any** solid food? (solid food includes things like baby cereal and pureed fruit but not drinks like formula or milk)
Please enter the number of days, weeks, months or years (choose one) or fill in the circle of the correct response.

[ ] days **OR** [ ] weeks **OR** [ ] months **OR** [ ] years **AND** [ ] months

- ○ Other, please explain: ______
- ○ Don't know

C.6 How old was the child when he/she first had solid food **regularly** (solid food includes things like baby cereal and pureed fruit but not drinks like formula or milk; regularly means more than twice a week for several continuous weeks)?
Please enter the number of days, weeks, months or years (choose one) or fill in the circle of the correct response.

[ ] days **OR** [ ] weeks **OR** [ ] months **OR** [ ] years **AND** [ ] months

- ○ Other, please explain: ______
- ○ Don't know

D. Your family's health history

D.1 Did your child have any issues with poor weight gain or feeding in the first two years of life?

○ Yes ○ No ○ Don't know

D.2 Is your child up to date with his/her immunisations (that is, needles or injections)? (fill in one circle only)

- ○ Yes, completely up to date
- ○ No, but has had most
- ○ No, but has had some
- ○ No, hasn't had any
- ○ Don't know

D.3 Have you ever been told by a doctor that your child has… (fill in one circle on each line)

No	Yes	
○	○	Diabetes
○	○	High blood pressure
○	○	High cholesterol or an abnormal amount of fat in their blood
○	○	Addison's disease
○	○	Asthma
○	○	Celiac disease
○	○	Hyperthyroidism (high thyroid)
○	○	Hypothyroidism (low thyroid)
○	○	Vitiligo (white skin patches)
○	○	Problems with his/her kidneys
○	○	Problems with his/her liver
○	○	Problems with his/her heart
○	○	Any condition not listed above (please specify ______

D.4 Has your child had any of the problems listed below? (fill in one circle on each line)

No	Yes	
○	○	Asthma needing medicine
○	○	Developmental delay
○	○	Mobility or joint pain
○	○	Repeated a grade
○	○	Operation(s). Please specify: ______
○	○	Other hospital admission(s). Please specify: ______
○	○	Other serious illnesses. Please specify: ______
○	○	Regularly takes prescribed medication(s). Please specify: ______
○	○	**Currently** taking prescribed or over-the-counter steroid medication(s) (this medication(s) can be either oral, topical or inhaled). Please specify: ______
○	○	**In the past** taken prescribed or over-the-counter steroid medication(s) (this medication(s) can be either oral, topical or inhaled). Please specify: ______
○	○	Allergies. Please specify: ______

D.5 Has anyone on the **mother's** side of this child's family had any of the problems listed below? (Fill in one circle for each person on each row)

	**Mother's** side of the family								
**Health Condition**	**Child's Mother**			**Child's Grandmother**			**Child's Grandfather**		
	**Yes**	**No**	**Don't know**	**Yes**	**No**	**Don't know**	**Yes**	**No**	**Don't know**
Is this person **biologically** related to the study child?	○	○	○	○	○	○	○	○	○
High blood pressure	○	○	○	○	○	○	○	○	○
Heart attack	○	○	○	○	○	○	○	○	○
Diabetes (if yes for child's mother, please complete Question D.6)	○	○	○	○	○	○	○	○	○
Stroke	○	○	○	○	○	○	○	○	○
Lap band or other surgery to reduce weight	○	○	○	○	○	○	○	○	○
Early (before puberty) severe obesity	○	○	○	○	○	○	○	○	○
High cholesterol or triglycerides	○	○	○	○	○	○	○	○	○
Polycystic ovary syndrome (PCOS)	○	○	○	○	○	○			
Thyroid problems	○	○	○	○	○	○	○	○	○

D.6 If you marked that the child's **mother** had diabetes, please mark which type of diabetes and the age when the doctor first told her that she had diabetes (fill in one circle on each line).

	**Yes**	**No**	**Don't know**	**If yes, please fill in the age when a doctor 1st said she had diabetes**
Type 1 diabetes	○	○	○	☐☐
Type 2 diabetes	○	○	○	☐☐
Unknown type	○	○	○	☐☐

D.7 Has anyone on the **father's** side of this child's family had any of the problems listed below? (Fill in one circle for each person on each row)

	**Father's** side of the family								
**Health Condition**	**Child's Father**			**Child's Grandmother**			**Child's Grandfather**		
	**Yes**	**No**	**Don't know**	**Yes**	**No**	**Don't know**	**Yes**	**No**	**Don't know**
Is this person **biologically** related to the study child?	O	O	O	O	O	O	O	O	O
High blood pressure	O	O	O	O	O	O	O	O	O
Heart attack	O	O	O	O	O	O	O	O	O
Diabetes (if yes for child's mother, please complete Question D.6)	O	O	O	O	O	O	O	O	O
Stroke	O	O	O	O	O	O	O	O	O
Lap band or other surgery to reduce weight	O	O	O	O	O	O	O	O	O
Early (before puberty) severe obesity	O	O	O	O	O	O	O	O	O
High cholesterol or triglycerides	O	O	O	O	O	O	O	O	O
Polycystic ovary syndrome (PCOS)				O	O	O			
Thyroid problems	O	O	O	O	O	O	O	O	O

D.8 If you reported that the study child's **father** had diabetes, please mark which type of diabetes and the age when the doctor first told him that he had diabetes (fill in one circle on each line).

	**Yes**	**No**	**Don't know**	**If yes, please fill in the age when a doctor 1st said he had diabetes**
Type 1 diabetes	O	O	O	☐☐
Type 2 diabetes	O	O	O	☐☐
Unknown type	O	O	O	☐☐

D.9 Currently, do you or anyone else usually smoke inside the house?

O Yes O No O Don't know

E. How your child spends his/her time

E.1. How much does your child enjoy physical activity? (fill in one circle only)

O Not at all O A bit O Quite a lot O A lot

E.2. Which of the following does your child have in his/her bedroom (fill in all circles that apply)

O Television O Computer O Internet access O None of these

E.3 Please fill in the information below for your child's current school, kinder or crèche(s). We will NOT be contacting the school/kinder/crèche.

Name of school	Postcode	Grade/Year level in school	# days attends	Distance from your house
School ______	☐☐☐☐	☐☐	☐☐	☐☐ km
Kinder ______	☐☐☐☐	☐☐	☐☐	☐☐ km
Creche/s ______	☐☐☐☐		☐☐	☐☐ km

Does your child attend kinder & crèche on any of the same days?

O Yes... number of days ☐

O No

Now, we are going to ask some questions about activities that your child does during a typical week.

E.4 Thinking about a typical week, how much time per day **OUTSIDE OF KINDER/CRECHE or SCHOOL HOURS** does your child usually spend in total doing the following activities: watching TV/DVD, working or playing on the computer, or playing video games on the computer or with a game system.

Please write in 0 if your child doesn't spend any time doing these activities. If your child spends some time (but less than one hour) doing these activities, write in '0' for the hours and then fill in the correct minutes.

	**Per day on creche/kinder/school days (outside of creche/kinder/school hours)**	**Per day on non-creche/kinder/school days**
Total time spent watching TV/DVD, working or playing on the computer, or playing video games on the computer or with a game system	☐☐ : ☐☐ hours minutes	☐☐ : ☐☐ hours minutes

E.5. Thinking about a typical week, how many hours and minutes per day does your child spend doing each of the following activities **OUTSIDE OF KINDER/CRECHE or SCHOOL HOURS?**

Please write in 0 if your child does not spend any time doing that activity. If your child spends some time (but less than one hour) doing an activity, write in '0' for the hours and then fill in the correct minutes.

	**Per day on creche/kinder/school days (outside of creche/kinder/school hours)**	**Per day on non-creche/kinder/school days**
a) Outdoors for transport (walking, biking, etc)?	☐☐ : ☐☐ hours minutes	☐☐ : ☐☐ hours minutes
b) Outdoors for play / recreation?	☐☐ : ☐☐ hours minutes	☐☐ : ☐☐ hours minutes
c) Watching TV or DVD?	☐☐ : ☐☐ hours minutes	☐☐ : ☐☐ hours minutes
d) On the computer (doing something other than games)?	☐☐ : ☐☐ hours minutes	☐☐ : ☐☐ hours minutes
e) Playing video games (either on the computer or using a game system like Nintendo)?	☐☐ : ☐☐ hours minutes	☐☐ : ☐☐ hours minutes

f) Please list the game systems that you play regularly

____

____

E.6 Thinking about a typical week, about how many days a week does…(fill in one circle on each line)

	Days per week 0	1	2	3	4	5	6	7
a) Your child eat breakfast?	O	O	O	O	O	O	O	O
b) Your family sit at a dinner table to eat the evening meal?	O	O	O	O	O	O	O	O
c) Your child eat a meal or snack in front of the TV?	O	O	O	O	O	O	O	O
d) Your child eat take-away meals (e.g., McDonalds, fish & chips, meats pies) (don't include school lunches)?	O	O	O	O	O	O	O	O
e) Your child watch TV/DVD in own room?	O	O	O	O	O	O	O	O
f) Your child do organised sport or physical activity (e.g., swimming, tennis, dance)?	O	O	O	O	O	O	O	O
g) Your child attend creche/kinder/school?	O	O	O	O	O	O		
h) Your child walk to or from creche/kinder/school?	O	O	O	O	O	O		
i) Your child bike/scooter to or from creche/kinder/school?	O	O	O	O	O	O		
j) Your child use public transportation?	O	O	O	O	O	O	O	O

E.7 Does anyone else play an important role in looking after this child? (please fill in one circle on each line)

Person	No	Yes	If yes, about how many hours per week?
Grandparent(s)	O	O	☐☐ hours per week
Parent who lives somewhere else	O	O	☐☐ hours per week
After-school or before-school program	O	O	☐☐ hours per week
Day care centre	O	O	☐☐ hours per week
Family day care	O	O	☐☐ hours per week
Preschool/kinder	O	O	☐☐ hours per week
Other person/s ____________ ____________	O	O	☐☐ hours per week

F. Your child's sleep

F.1. About what time does your child usually... (please circle am or pm)

	Creche/Kinder/School day	Non-Creche/Kinder/School day
Go to bed at night?	__ __ : __ __ am / pm	__ __ : __ __ am / pm
Go to sleep at night?	__ __ : __ __ am / pm	__ __ : __ __ am / pm
Wake up in the morning?	__ __ : __ __ am / pm	__ __ : __ __ am / pm

F.2. Does your child go to bed at regular times? (fill in one circle only)

○ Always ○ Usually ○ Sometimes ○ Rarely ○ Never

F.3 How much is your child's sleeping pattern or habits a problem for you? (fill in one circle only)

○ A large problem ○ A moderate problem ○ A small problem ○ No problem at all

F.4 Does your child have any of these problems on 4 or more nights a week, that is, more than half the time? (fill in one circle on each line).

Problem	Yes	No	Don't know
a) Difficulty getting off to sleep at night?	○	○	○
b) Not happy to sleep alone?	○	○	○
c) Waking during the night?	○	○	○
d) Restless sleep?	○	○	○
e) Bed wetting?	○	○	○
f) Nightmares, night terrors?	○	○	○
g) Wheezing or asthma?	○	○	○
h) Snoring or difficulty breathing?	○	○	○
i) Seeming tired in the morning?	○	○	○
j) Other problem (please describe)	○	○	○

____

____

G. Your neighbourhood

G.1 Now, some questions about where your child's plays? (fill in one circle on each line)

	Yes	No	
a) Do you have a safe front or back yard where your child can play?	○	○	→ *go to Question G.1 c)*
b) **If yes**, do you need to be physically present when he/she plays there?	○	○	
c) Does your child have access to other safe play areas (eg, local park)?	○	○	
d) Does your family own a car?	○	○	

G.2. What do you think of your neighbourhood as a place to live? (fill in one circle only)

○ A very good place to live
○ A fairly good place to live
○ Not a very good place to live
○ Not a very good place to live at all

G.3. How do you feel about your neighbourhood as a place to bring up children? (fill in one circle only)

○ Very good
○ Good
○ Fair
○ Poor
○ Very poor

G.4. How long have you lived in this neighbourhood?

☐☐ months **OR** ☐☐ years

G.5What is your home postcode?

☐☐☐☐

G.6. Now, I'm going to ask how strongly you agree or disagree with these statements about your neighbourhood? For each, mark whether you 'strongly agree', 'agree', 'disagree', 'strongly disagree' or 'don't know'. Please answer each question (fill in one circle on each line).

	Strongly disagree	Disagree	Agree	Strongly agree	Don't know
a) This is a safe neighbourhood	○	○	○	○	○
b) This is a clean neighbourhood	○	○	○	○	○
c) There are good parks, playgrounds and play spaces in this neighbourhood.	○	○	○	○	○
d) There is good street lighting in this neighbourhood.	○	○	○	○	○
e) The state of the footpaths and roads is good in this neighbourhood.	○	○	○	○	○
f) There is access to close, affordable, regular public transport in this neighbourhood.	○	○	○	○	○

	Strongly disagree	Disagree	Agree	Strongly agree	Don't know
g) There is access to basic shopping facilities in this neighbourhood.	O	O	O	O	O
h) There is access to basic services such as banks, medical clinics, etc in this neighbourhood	O	O	O	O	O
i) There is heavy traffic in my street or road.	O	O	O	O	O
j) It is safe for children to play outside during the day.	O	O	O	O	O
k) People around here are willing to help their neighbours.	O	O	O	O	O

G.7. Now, we are going to ask some specific questions about how you think about your neighbourhood. Both 'local' and 'within walking distance' mean within a 10-15 minute walk from your home.

	Strongly disagree	Disagree	Agree	Strongly agree
a) Stores are within easy walking distance of my home.	O	O	O	O
b) There are many places to go within easy walking distance of my home.	O	O	O	O
c) It is easy to walk to a transit stop (bus, train, tram) from my home.	O	O	O	O
d) The distance between intersections in my neighbourhood is usually short (100 metres or less)	O	O	O	O
e) There are many alternative routes for getting from place to place in my neighbourhood (I don't have to go the same way every time).	O	O	O	O
f) There are footpaths on most of the streets in my neighbourhood.	O	O	O	O
g) Footpaths are separated from the road/traffic in my neighbourhood by parked cars.	O	O	O	O
h) There is a grass/dirt strip that separates the streets from the footpaths in my neighbourhood.	O	O	O	O
i) My neighbourhood streets are well lit at night.	O	O	O	O
j) Walkers and bikers on the streets in my neighbourhood can be easily seen by people in their homes.	O	O	O	O
k) There are crosswalks and pedestrian signals to help walkers cross busy streets in my neighbourhood.	O	O	O	O
l) There are trees along the streets in my neighbourhood.	O	O	O	O

	Strongly disagree	Disagree	Agree	Strongly agree
m) There are many interesting things to look at while walking in my neighbourhood.	O	O	O	O
n) There are many attractive natural sights in my neighbourhood	O	O	O	O
o) There are attractive buildings/homes in my neighbourhood.	O	O	O	O
p) There is so much traffic along **nearby** streets that it makes it difficult or unpleasant to walk in my neighbourhood.	O	O	O	O
q) The speed of traffic on most **nearby** streets is usually slow (50 km/hr or less).	O	O	O	O
r) Most drivers exceed the posted speed limits while driving in my neighbourhood.	O	O	O	O
s) There is a high crime rate in my neighbourhood.	O	O	O	O
t) The crime rate in my neighbourhood makes it unsafe to go on walks **during the day**.	O	O	O	O
u) The crime rate in my neighbourhood makes it unsafe to go on walks **at night**.	O	O	O	O

H. Information about your household

Now, we are going to ask some questions specifically about the parents that **live in this household.**

H.1 Do you have a **spouse/partner** who **currently lives** with you and your child?

O No → *Only indicate responses in the 'Person 1' column*

O Yes → *Please complete **both** the 'Person 1' and 'Person 2' columns*

	**Person 1**<br>**Primary Caregiver**	**Person 2**<br>**Partner/Spouse**
H.2 Date of birth	☐☐ / ☐☐ / ☐☐☐☐<br>day month year	☐☐ / ☐☐ / ☐☐☐☐<br>day month year
H.3 Gender	O Male<br>O Female	O Male<br>O Female
H.4 What is the person's relationship to person 1?	No answer required for person 1	O Husband or wife of person 1<br>O De facto partner of person 1<br>O Other (please specify)<br>__________

	Person 1<br>Primary Caregiver	Person 2<br>Partner/Spouse
H.5 What is the person's present marital status?	○ Never married<br>○ Widowed<br>○ Divorced<br>○ Separated but not divorced<br>○ Married	○ Never married<br>○ Widowed<br>○ Divorced<br>○ Separated but not divorced<br>○ Married
H.6 How is this person related to the child visiting the Weight Management Service doctors today?	○ Biological parent<br>○ Step parent<br>○ Other, please specify<br>__________	○ Biological parent<br>○ Step parent<br>○ Other, please specify<br>__________
H.7 What is this person's height?	☐☐☐ centimeters	☐☐☐ centimeters
H.8 What is this person's weight?	☐☐☐.☐ kilos	☐☐☐.☐ kilos
H.9 In which country was the person born?	○ Australia<br>○ Other, please specify<br>__________	○ Australia<br>○ Other, please specify<br>__________
H.10 If other, enter the year when the person first arrived to live in Australia for 1 year or more	☐☐☐☐<br>year	☐☐☐☐<br>year
H.11 Is this person of Aboriginal or Torres Strait Islander origin?	○ No<br>○ Yes, Aboriginal<br>○ Yes, Torres Strait Islander<br>○ Yes, Aboriginal & Torres Strait Islander<br>○ Don't know	○ No<br>○ Yes, Aboriginal<br>○ Yes, Torres Strait Islander<br>○ Yes, Aboriginal & Torres Strait Islander<br>○ Don't know
H.12 Does this person speak a language other than English **in the home?**	○ No, English only<br>○ Yes, Italian<br>○ Yes, Greek<br>○ Yes, Cantonese<br>○ Yes, Arabic<br>○ Yes, Vietnamese<br>○ Yes, Mandarin<br>○ Yes, Other (please specify)<br>__________	○ No, English only<br>○ Yes, Italian<br>○ Yes, Greek<br>○ Yes, Cantonese<br>○ Yes, Arabic<br>○ Yes, Vietnamese<br>○ Yes, Mandarin<br>○ Yes, Other (please specify)<br>__________

continued...

	Person 1<br>Primary Caregiver	Person 2<br>Partner/Spouse
H.13 What is this persons ancestry? Provide up to 2 ancestries only, Examples of 'other' include: Greek, Vietnamese, Hmong, Dutch, Kurdish, Maori, Lebanese, Australian South Sea Islander	○ English<br>○ Irish<br>○ Italian<br>○ German<br>○ Chinese<br>○ Scottish<br>○ Australian<br>○ Other, please specify<br>__________	○ English<br>○ Irish<br>○ Italian<br>○ German<br>○ Chinese<br>○ Scottish<br>○ Australian<br>○ Other, please specify<br>__________
H.14 What was the highest year of primary or secondary school this person completed?	○ Year 12 or equivalent<br>○ Year 11 or equivalent<br>○ Year 10 or equivalent<br>○ Year 9 or equivalent<br>○ Year 8 or below<br>○ Never attended school<br>○ Still at school<br>○ Don't know	○ Year 12 or equivalent<br>○ Year 11 or equivalent<br>○ Year 10 or equivalent<br>○ Year 9 or equivalent<br>○ Year 8 or below<br>○ Never attended school<br>○ Still at school<br>○ Don't know
H.15 Has this person completed a trade certificate or any other educational qualification?	○ No<br>○ No, still studying for first qualification<br>○ Yes, trade certificate/ apprenticeship<br>○ Yes, other qualification<br>○ Don't know	○ No<br>○ No, still studying for first qualification<br>○ Yes, trade certificate/ apprenticeship<br>○ Yes, other qualification<br>○ Don't know
H.16 What is the highest qualification that this person completed? (Bachelor Degree includes Honours)	○ Postgraduate degree<br>○ Graduate diploma/certificate<br>○ Bachelor degree<br>○ Advanced diploma/diploma<br>○ Certificate<br>○ Other<br>__________<br>○ Don't know	○ Postgraduate degree<br>○ Graduate diploma/certificate<br>○ Bachelor degree<br>○ Advanced diploma/diploma<br>○ Certificate<br>○ Other<br>__________<br>○ Don't know
H.17 What is this person's occupation?	__________<br>occupation	__________<br>occupation

continued...

	Person 1<br>Primary Caregiver	Person 2<br>Partner/Spouse
H.18 What is this person's current employment status?	○ Full-time paid employment<br>○ Part-time paid employment<br>○ Unemployed, seeking work<br>○ Not in paid labour force (e.g. retired, parenting full time)<br>○ Student<br>○ Other, please specify ________	○ Full-time paid employment<br>○ Part-time paid employment<br>○ Unemployed, seeking work<br>○ Not in paid labour force (e.g. retired, parenting full time)<br>○ Student<br>○ Other, please specify ________
H.19 If you are in paid employment, about how many hours per week do you work?	☐☐☐ hours per week	☐☐☐ hours per week
H.20 What is the total of all wages/salaries, government benefits, pensions, allowances and other income this person usually received?	○ $2400 or more per week ($124,800 per year)<br>○ $2200-$2399 per week ($114,400-$124,799 per year)<br>○ $2000-$2199 per week ($104,000-$114,399 per year)<br>○ $1600-$1999 per week ($83,200-$103,999 per year)<br>○ $1300-$1599 per week ($67,600-$83,199 per year)<br>○ $1000-$1299 per week ($52,000-$67,599 per year)<br>○ $800-$999 per week ($41,600-$51,999 per year)<br>○ $600-$799 per week ($31,200-$41,599 per year)<br>○ $400-$599 per week ($20,800-$31,199 per year)<br>○ $250-$399 per week ($13,000-$20,799 per year)<br>○ $150-$249 per week ($7,800-$12,999 per year)<br>○ $1-$149 per week ($1-$7,799 per year)<br>○ No income<br>○ Negative income	○ $2400 or more per week ($124,800 per year)<br>○ $2200-$2399 per week ($114,400-$124,799 per year)<br>○ $2000-$2199 per week ($104,000-$114,399 per year)<br>○ $1600-$1999 per week ($83,200-$103,999 per year)<br>○ $1300-$1599 per week ($67,600-$83,199 per year)<br>○ $1000-$1299 per week ($52,000-$67,599 per year)<br>○ $800-$999 per week ($41,600-$51,999 per year)<br>○ $600-$799 per week ($31,200-$41,599 per year)<br>○ $400-$599 per week ($20,800-$31,199 per year)<br>○ $250-$399 per week ($13,000-$20,799 per year)<br>○ $150-$249 per week ($7,800-$12,999 per year)<br>○ $1-$149 per week ($1-$7,799 per year)<br>○ No income<br>○ Negative income

	Person 1<br>Primary Caregiver	Person 2<br>Partner/Spouse
H.21 Other than the people you've already told us about in question H.20, are there any other people living in the household that are age 15 years or older and are employed?	○ No<br>○ Yes → Please tell us their **annual** income using the income amounts on the previous page<br>__________<br>__________<br>__________	
H.22 Does this person currently smoke?	○ Yes<br>○ No	○ Yes<br>○ No
H.23 Has this person ever smoked regularly (that is, at least once a day)?	○ Yes<br>○ No	○ Yes<br>○ No

I. Other family members

I.1 How many people regularly live in your household? [ ][ ]

I.2 Does the child visiting the Weight Management Service today regularly spend time living in another household (for example, if his/her parents are divorced, does the study child spend time living in another household)?

○ No (child usually spends 7 days per week in this household)
○ Yes (please tell us the amount spent in this household below)

- ○ 6 days per week
- ○ 5 days per week
- ○ 4 days per week
- ○ 3 days per week
- ○ 2 days per week
- ○ 1 day per week
- ○ 1 day per fortnight
- ○ 2 days per fortnight
- ○ Other (please explain) __________

I.3 Do any of this child's siblings have any medical illness? (please explain)

____

____

I.4 Do any of this child's siblings have weight issues? (please explain)

____

____

This page has been left blank intentionally.

Lastly, for your child's brothers and sisters and the people that live in your household most of the time, we would like to know their date of birth, gender and their relationship to your child visiting the Weight Management Service. Please be as specific as possible in describing the relationship (e.g. mother, step-mother, brother, sister, half-brother (same mother), half-sister (same father), step-sister, etc).

**Given name**	**Relationship to child visiting service**	**Date of birth**	**Gender**	**Does this person regularly live in the household**
______ ______	______ ______	__ __ / __ __ / __ __ __ __ (day / month / year)	O Male O Female	O Yes O No
______ ______	______ ______	__ __ / __ __ / __ __ __ __ (day / month / year)	O Male O Female	O Yes O No
______ ______	______ ______	__ __ / __ __ / __ __ __ __ (day / month / year)	O Male O Female	O Yes O No
______ ______	______ ______	__ __ / __ __ / __ __ __ __ (day / month / year)	O Male O Female	O Yes O No
______ ______	______ ______	__ __ / __ __ / __ __ __ __ (day / month / year)	O Male O Female	O Yes O No
______ ______	______ ______	__ __ / __ __ / __ __ __ __ (day / month / year)	O Male O Female	O Yes O No
______ ______	______ ______	__ __ / __ __ / __ __ __ __ (day / month / year)	O Male O Female	O Yes O No
______ ______	______ ______	__ __ / __ __ / __ __ __ __ (day / month / year)	O Male O Female	O Yes O No

continued...

Given name	Relationship to child visiting service	Date of birth	Gender	Does this person regularly live in the household
		☐☐ / ☐☐ / ☐☐☐☐ day month year	○ Male ○ Female	○ Yes ○ No
		☐☐ / ☐☐ / ☐☐☐☐ day month year	○ Male ○ Female	○ Yes ○ No
		☐☐ / ☐☐ / ☐☐☐☐ day month year	○ Male ○ Female	○ Yes ○ No
		☐☐ / ☐☐ / ☐☐☐☐ day month year	○ Male ○ Female	○ Yes ○ No
		☐☐ / ☐☐ / ☐☐☐☐ day month year	○ Male ○ Female	○ Yes ○ No
		☐☐ / ☐☐ / ☐☐☐☐ day month year	○ Male ○ Female	○ Yes ○ No
		☐☐ / ☐☐ / ☐☐☐☐ day month year	○ Male ○ Female	○ Yes ○ No

Participant code: ☐☐☐☐☐☐☐☐☐☐☐☐☐

J. Alternate contact details

During these types of studies, families sometimes move house. It's very helpful to have an extra contact person, so we can stay in touch even if you move unexpectedly. We'd appreciate it if you could fill in the following alternate contact details. All information you give us will be kept strictly confidential.

Name: ______________________

Relationship to child/family: ______________________

Address: ______________________

Suburb: ______________________ **Postcode:** ☐☐☐☐

Telephone: (BH) ______________ **(AH)** ______________

Mobile: ______________________

Email: ______________________

Mailing address (if different from above):

Address: ______________________

Suburb: ______________________ **Postcode:** ☐☐☐☐

Please check that you have answered all questions on both sides of each page.

A researcher will meet you at your visit to answer any questions and to collect the completed survey.

Thank you for your participation!

This page has been left blank intentionally.
