# Supplementary material for: New directions in childhood obesity research: how a comprehensive biorepository will allow better prediction of outcomes
Source: BMC Med Res Methodol. 2010 Oct 22;10:100. doi: 10.1186/1471-2288-10-100 (PMC2984501; doi:10.1186/1471-2288-10-100)
Supplement: Additional file 6 — F. COBRA Survey 1 Teens 14yo.pdf. COBRA Survey 1 for teens aged 14 years and older [file 1471-2288-10-100-S6.PDF]

9543081325

Participant code: | | | | | | | | | | | | | |

COBRA
Childhood Overweight
BioRepository of Australia

Survey 1 (TEENS)

This survey is for teens age 14 years and older

Please bring the completed survey with you when you come to your next visit at The Royal Children's Hospital Weight Management Service.

You will give it to the researcher who meets you at this visit.

Thank you for agreeing to be in this study. This study is looking at things that may affect health problems for some overweight or obese children.

Before your first visit, we would like to get an idea about your general health and some idea about your activities and your neighbourhood. Please answer the questions on the next pages.

This should take you about 15-20 minutes.

It is private, and your answers are confidential.

INSTRUCTIONS

1. Please answer by filling in the circles completely like this ●
2. If you make a mistake, put a cross through it, then fill in and draw a circle around the correct one.
3. Use a blue or black pen only.
4. There are no right or wrong answers. If you aren't sure, just give the best answer you can. You can also make a comment in the margin - it will be read!
5. Please remember to fill in the back of each page as well.

Questions? Ring us (03) 9936 6512 or email us (mpowr@mcri.edu.au) any time

Thank you for taking part in COBRA

OFFICE USE ONLY

Date returned: __ __ / __ __ / __ __ __ __

Was survey completed before seeing clinicans? ○ No ○ Yes

Completed at: ○ RCH ○ Home ○ Other ____________

Murdoch Childrens Research Institute
Healthier Kids. Healthier Future.

The Royal Children's Hospital Melbourne

A. General health

A.1. Today's date: ☐☐ / ☐☐ / ☐☐☐☐

day month year

A.2. In general, would you say your health is (fill in one circle only)

○ Excellent ○ Very good ○ Good ○ Fair ○ Poor

A.3. How concerned are **you** about your current weight? (fill in one circle only)

○ Not at all ○ A little ○ Moderately ○ Very

A.4. How concerned do you think **your parents** are about your current weight? (fill in one circle only)

○ Not at all ○ A little ○ Moderately ○ Very

B. Your behaviour and emotions

For each item, please fill in the circle for 'Not True', 'Somewhat True' or 'Certainly True'. It would help us if you answered all items as best you can even if you are not absolutely certain. Please give your answers on the basis of how things have been for you over the last **six months. Fill in one circle on each line.**

	**Not true**	**Somewhat true**	**Certainly true**
a) I try to be nice to other people. I care about their feelings.	○	○	○
b) I am restless, I cannot stay still for long.	○	○	○
c) I get a lot of headaches, stomach-aches, or sickness.	○	○	○
d) I usually share with others, for example CDs, games, food.	○	○	○
e) I get very angry and often lose my temper.	○	○	○
f) I would rather be alone than with people of my age.	○	○	○
g) I usually do as I am told.	○	○	○
h) I worry a lot.	○	○	○
i) I am helpful if someone is hurt, upset or feeling ill.	○	○	○
j) I am constantly fidgeting or squirming.	○	○	○
k) I have one good friend or more.	○	○	○
l) I fight a lot. I can make other people do what I want.	○	○	○
m) I am often unhappy, depressed or tearful.	○	○	○
n) Other people my age generally like me.	○	○	○

...continued

	Not true	Somewhat true	Certainly true
o) I am easily distracted, I find it difficult to concentrate.	○	○	○
p) I am nervous in new situations. I easily lose confidence.	○	○	○
q) I am kind to younger children.	○	○	○
r) I am often accused of lying or cheating.	○	○	○
s) Other children or young people pick on me or bully me.	○	○	○
t) I often volunteer to help others (parents, teachers, children).	○	○	○
u) I think before I do things.	○	○	○
v) I take things that are not mine from home, school or elsewhere.	○	○	○
w) I get along better with adults than with people my own age.	○	○	○
x) I have many fears, I am easily scared.	○	○	○
y) I finish the work I'm doing. My attention is good.	○	○	○

SDQ S11-17 © Copyright Robert Goodman, 2005

C. How you spend your time

C.1. How much do you enjoy physical activity? (fill in one circle only)

○ Not at all ○ A bit ○ Quite a lot ○ A lot

C.2. Please mark all the things listed below that you have in your bedroom (fill in all circles that apply)

○ Television ○ Computer ○ Internet access ○ None of these

C.3. Thinking about a typical week, how much time per day **OUTSIDE OF SCHOOL HOURS** do you usually spend in total doing the following activities: watching TV/DVD, working or playing on the computer, or playing video games on the computer or with a game system.

Please write in 0 if you do not spend any time doing these activities. If you spend some time (but less than one hour) doing these activities, write in '0' for the hours and then fill in the correct minutes.

	**Per day on school days (outside of school hours)**	**Per day on non-school days**
Total time spent watching TV/DVD, working or playing on the computer, or playing video games on the computer or with a game system	☐☐ : ☐☐<br>hours minutes	☐☐ : ☐☐<br>hours minutes

C.4. Thinking about a typical week, how many hours and minutes per day do you spend doing each of the following activities **OUTSIDE OF SCHOOL HOURS?**

Please write in 0 if you do not spend any time doing that activity. If you spend some time (but less than one hour) doing an activity, write in '0' for the hours and then fill in the correct minutes.

	**Per day on school days (outside of school hours)**	**Per day on non-school days**
a) Outdoors for transport (walking, biking, etc)?	☐☐ : ☐☐ hours minutes	☐☐ : ☐☐ hours minutes
b) Outdoors for play / recreation?	☐☐ : ☐☐ hours minutes	☐☐ : ☐☐ hours minutes
c) Watching TV or DVD?	☐☐ : ☐☐ hours minutes	☐☐ : ☐☐ hours minutes
d) On the computer (doing something other than games)?	☐☐ : ☐☐ hours minutes	☐☐ : ☐☐ hours minutes
e) Playing video games (either on the computer or using a game system like Nintendo)?	☐☐ : ☐☐ hours minutes	☐☐ : ☐☐ hours minutes

f) Please list the game systems that you play regularly

____

____

C.5. Thinking about a typical week, about how many days a week do you / does your family…(fill in one circle on each line)

	Days per week							
	**0**	**1**	**2**	**3**	**4**	**5**	**6**	**7**
a) You eat breakfast?	O	O	O	O	O	O	O	O
b) Your family sit at a dinner table to eat the evening meal?	O	O	O	O	O	O	O	O
c) You eat a meal or snack in front of the TV?	O	O	O	O	O	O	O	O
d) You eat take-away meals (e.g., McDonalds, fish & chips, meats pies) (don't include school lunches)?	O	O	O	O	O	O	O	O
e) You watch TV/DVD in your own room?	O	O	O	O	O	O	O	O
f) You do organised sport or physical activity (e.g., swimming, tennis, dance)?	O	O	O	O	O	O	O	O
g) You attend school?	O	O	O	O	O	O		
h) You walk to or from school?	O	O	O	O	O	O		
i) You bike/scooter to or from school?	O	O	O	O	O	O		
j) You use public transportation?	O	O	O	O	O	O	O	O

C.6. Now, we are going to ask some specific questions about activities **over the past 7 days**. Please fill in the number of days and usual amount of time on one day (include hours AND minutes) on each line; write '0' if you don't do that activity.

	**Number of days**	**Time usually spent on ONE of those days**
During the last 7 days, on how many days did you travel in a motor vehicle like a train, bus, car or tram?	☐	☐☐ : ☐☐ hours minutes
**Now, think only of bicycling and walking you might have done to travel to and from school, to do errands, or to go from place to place.**		
During the **last 7 days**, on how many days did you **bicycle** for at least 10 minutes at a time **to go from place to place**?	☐	☐☐ : ☐☐ hours minutes
During the **last 7 days**, on how many days did you **walk** for at least 10 minutes at a time **to go from place to place**?	☐	☐☐ : ☐☐ hours minutes
Not counting any walking you have already mentioned, during the **last 7 days**, on how many days did you walk for at least 10 minutes at a time **in your leisure time**?	☐	☐☐ : ☐☐ hours minutes

D. Your sleep

D.1. About what time do you usually... (please circle am or pm)

	School day	Non-school day
Go to bed at night?	__ __ : __ __ am / pm	__ __ : __ __ am / pm
Go to sleep at night?	__ __ : __ __ am / pm	__ __ : __ __ am / pm
Wake up in the morning?	__ __ : __ __ am / pm	__ __ : __ __ am / pm

D.2. Do you go to bed at regular times? (fill in one circle only)

O Always O Usually O Sometimes O Rarely O Never

D.3. During the **past month**, how would you rate your sleep **quality** overall (how **well** you sleep)? (fill in one circle only)

O Very good O Fairly good O Good O Fairly bad O Very bad

D.4. During the **past month**, how would you rate your sleep **quantity** overall (how **much** you sleep)? (fill in one circle only)

O Very good O Fairly good O Good O Fairly bad O Very bad

E. About your neighbourhood

E.1. What do you think of your neighbourhood as a place to live? (fill in one circle only)

O A very good place to live O A fairly good place to live O Not a very good place to live O Not a very good place to live at all

E.2. How do you feel about your neighbourhood as a place to bring up children? (fill in one circle only)

O Very good O Good O Fair O Poor O Very poor

E.3. Now, I'm going to ask how strongly you agree or disagree with these statements about your neighbourhood? For each, mark whether you 'strongly agree', 'agree', 'disagree', 'strongly disagree' or 'don't know'. Please answer each question (fill in one circle on each line).

	Strongly disagree	Disagree	Agree	Strongly agree	Don't know
a) This is a safe neighbourhood	○	○	○	○	○
b) This is a clean neighbourhood	○	○	○	○	○
c) There are good parks, playgrounds and play spaces in this neighbourhood.	○	○	○	○	○
d) There is good street lighting in this neighbourhood.	○	○	○	○	○
e) The footpaths and roads in this neighbourhood are in good shape.	○	○	○	○	○
f) It is easy to get to public transportation like trains or trams in this neighbourhood.	○	○	○	○	○
g) This neighbourhood has basic shops for things like bread and milk.	○	○	○	○	○
h) This neighbourhood has services like banks and doctors.	○	○	○	○	○
i) There is heavy traffic in my street or road.	○	○	○	○	○
j) It is safe for children to play outside during the day.	○	○	○	○	○
k) People around here are willing to help their neighbours.	○	○	○	○	○

E.4. Now, we are going to ask some specific questions about how you think about your neighbourhood. Both 'local' and 'within walking distance' mean within a 10-15 minute walk from your home.

	Strongly disagree	Disagree	Agree	Strongly agree
a) Stores are within easy walking distance of my home.	○	○	○	○
b) There are many places to go within easy walking distance of my home.	○	○	○	○
c) It is easy to walk to a transit stop (bus, train, tram) from my home.	○	○	○	○
d) The distance between intersections in my neighbourhood is usually short (100 metres or less)	○	○	○	○
e) There are many alternative routes for getting from place to place in my neighbourhood (I don't have to go the same way every time).	○	○	○	○
f) There are footpaths on most of the streets in my neighbourhood.	○	○	○	○

...continued

	Strongly disagree	Disagree	Agree	Strongly agree
g) Footpaths are separated from the road/traffic in my neighbourhood by parked cars.	○	○	○	○
h) There is a grass/dirt strip that separates the streets from the footpaths in my neighbourhood.	○	○	○	○
i) My neighbourhood streets are well lit at night.	○	○	○	○
j) Walkers and bikers on the streets in my neighbourhood can be easily seen by people in their homes.	○	○	○	○
k) There are crosswalks and pedestrian signals to help walkers cross busy streets in my neighbourhood.	○	○	○	○
l) There are trees along the streets in my neighbourhood.	○	○	○	○
m) There are many interesting things to look at while walking in my neighbourhood.	○	○	○	○
n) There are many attractive natural sights in my neighbourhood (such as landscaping, views)	○	○	○	○
o) There are attractive buildings/homes in my neighbourhood.	○	○	○	○
p) There is so much traffic along **nearby** streets that it makes it difficult or unpleasant to walk in my neighbourhood.	○	○	○	○
q) The speed of traffic on most **nearby** streets is usually slow (50 km/hr or less).	○	○	○	○
r) Most drivers exceed the posted speed limits while driving in my neighbourhood.	○	○	○	○
s) There is a high crime rate in my neighbourhood.	○	○	○	○
t) The crime rate in my neighbourhood makes it unsafe to go on walks **during the day**.	○	○	○	○
u) The crime rate in my neighbourhood makes it unsafe to go on walks **at night**.	○	○	○	○

Please check that you have answered all questions on both sides of each page.
A researcher will meet you at your visit to answer any questions
and to collect the completed survey.

Thank you for your participation!
