# Supplementary material for: New directions in childhood obesity research: how a comprehensive biorepository will allow better prediction of outcomes
Source: BMC Med Res Methodol. 2010 Oct 22;10:100. doi: 10.1186/1471-2288-10-100 (PMC2984501; doi:10.1186/1471-2288-10-100)
Supplement: Additional file 7 — G. COBRA Survey 2 Parent 1yo.pdf. COBRA Survey 2 for parents of 1 year olds [file 1471-2288-10-100-S7.PDF]

2357218761

Participant code: | | | | | | | | | | | | | |

COBRA

Childhood Overweight
BioRepository of Australia

Survey 2 (PARENT)

This form is for the parents of children age 1 year

Thank you for being in this study. This study is looking at the things that may affect health problems for some overweight or obese children.

Before your child sees the doctors, we would like to get an idea about how you have been feeling. You can talk about the questions with your child's doctor **after** you are finished. A researcher is here to help you if you have any questions.

This should take you about 5 minutes.

It is private, and your answers are confidential.

INSTRUCTIONS

1. Please answer by filling in the circles completely like this ●
2. If you make a mistake, put a cross through it, then fill in and draw a circle around the correct one.
3. Use a blue or black pen only.
4. There are no right or wrong answers. If you aren't sure, just give the best answer you can. You can also make a comment in the margin - it will be read!
5. Please remember to fill in the back of each page as well.

When you are done, please give this survey to the researcher working with you today.

Questions? Ring us (03) 9936 6512 or email us (mpowr@mcri.edu.au) any time

Thank you for taking part in COBRA

OFFICE USE ONLY

Date returned: ☐☐ / ☐☐ / ☐☐☐☐

Was survey completed before seeing clinicans? ○ No ○ Yes

Completed at: ○ RCH ○ Home ○ Other ____________

Murdoch Childrens Research Institute
Healthier Kids. Healthier Future.

The Royal Children's Hospital Melbourne

A. How <u>you</u> have been feeling

A.1. Who is completing this form? Fill in one circle only

○ Biological mother ○ Biological father ○ Other, please specify

Now we would like to know a bit more about how **YOU** have been feeling. If you are worried about anything, be sure to talk about it with your doctor.

Remember the questions below are about you, **not** your child.

Now we'd like you to think about how **you** have been feeling during the **past month**. For each question, please fill in one circle that best describes how often you had this feeling.

During the **past month**, how often did **you** feel...

	**None** of the time	**A little** of the time	**Some** of the time	**Most** of the time	**All** of the time
**a.** ...tired out for no good reason	○	○	○	○	○
**b.** ...nervous	○	○	○	○	○
**c.** ... so nervous that nothing could calm you down	○	○	○	○	○
**d.** ... hopeless	○	○	○	○	○
**e.** ... restless or fidgety	○	○	○	○	○
**f.** ... so restless that you could not sit still	○	○	○	○	○
**g.** ... depressed	○	○	○	○	○
**h.** ... so depressed that nothing could cheer you up	○	○	○	○	○
**i.** ... that everything was an effort	○	○	○	○	○
**j.** ... worthless	○	○	○	○	○

K10 Kessler et al (2003)

Please check that you have answered all questions on both sides of each page. Return to the researcher who is working with you today.

Thank you for your participation!
