# Supplementary material for: New directions in childhood obesity research: how a comprehensive biorepository will allow better prediction of outcomes
Source: BMC Med Res Methodol. 2010 Oct 22;10:100. doi: 10.1186/1471-2288-10-100 (PMC2984501; doi:10.1186/1471-2288-10-100)
Supplement: Additional file 11 — K. COBRA Survey 2 Parent 13yo.pdf. COBRA Survey 1 for parents of youth aged 13 years and older [file 1471-2288-10-100-S11.PDF]

1346372387

Participant code: | | | | | | | | | | | | | |

Survey 2 (PARENT)

This form is for the parents of teens age 13 years and older

Thank you for being in this study. This study is looking at the things that may affect health problems for some overweight or obese children.

Before you see the doctors, we would like to get an idea about your child's general health and well-being and how you feel. Please answer the questions on the next pages. Your child is completing similar questions. Please do not help your child with their answers (you can talk about the questions with your child or doctor **after** you both are finished). A researcher is here to help you if you have any questions.

This should take you about 10 minutes.

It is private, and your answers are confidential.

INSTRUCTIONS

1. Please answer by filling in the circles completely like this ●
2. If you make a mistake, put a cross through it, then fill in and draw a circle around the correct one.
3. Use a blue or black pen only.
4. There are no right or wrong answers. If you aren't sure, just give the best answer you can. You can also make a comment in the margin - it will be read!
5. Please remember to fill in the back of each page as well.

When you are done, please give this survey to the researcher working with you today.

Questions? Ring us (03) 9936 6512 or email us (mpowr@mcri.edu.au) any time

Thank you for taking part in COBRA

OFFICE USE ONLY

Date returned: ☐☐ / ☐☐ / ☐☐☐☐

Was survey completed before seeing clinicans? ○ No ○ Yes

Completed at: ○ RCH ○ Home ○ Other ____________

A. Your child's health and well-being

A.1. Who is completing this form? Fill in one circle only

○ Biological mother ○ Biological father ○ Other, please specify

Below is a list of things that might be a problem for **your teen**. Please tell us **how much of a problem** each one has been for **your teen** during the **past ONE month** by filling in the circle under the colums if the problem is either:

- **never** a problem
- **almost never** a problem
- **sometimes** a problem
- **often** a problem
- **almost always** a problem

There are no right or wrong answers. If you do not understand a question, please ask for help.

In the **past ONE month**, how much of a **problem** has this been for your teen?

Physical Functioning (problems with...)	Never	Almost Never	Some-times	Often	Almost Always
a. Walking more than 100 metres	○	○	○	○	○
b. Running	○	○	○	○	○
c. Participating in sports activity or exercise	○	○	○	○	○
d. Lifting something heavy	○	○	○	○	○
e. Taking a bath or shower by him or herself	○	○	○	○	○
f. Doing chores around the house	○	○	○	○	○
g. Having aches or pains	○	○	○	○	○
h. Having a low energy level	○	○	○	○	○

Emotional Functioning (problems with...)	Never	Almost Never	Some-times	Often	Almost Always
i. Feeling afraid or scared	○	○	○	○	○
j. Feeling sad	○	○	○	○	○
k. Feeling angry	○	○	○	○	○
l. Having trouble sleeping	○	○	○	○	○
m. Worrying about what will happen to him or her	○	○	○	○	○

Social Functioning (problems with...)	Never	Almost Never	Some-times	Often	Almost Always
n. Getting along with other teens	○	○	○	○	○
o. Other teens not wanting to be his or her friend	○	○	○	○	○
p. Getting teased by other teens	○	○	○	○	○
q. Not being able to do things that other teens his or her age can do	○	○	○	○	○
r. Keeping up with other teens	○	○	○	○	○

School Functioning (problems with...)	Never	Almost Never	Some-times	Often	Almost Always
s. Paying attention in class	○	○	○	○	○
t. Forgetting things	○	○	○	○	○
u. Keeping up with schoolwork	○	○	○	○	○
v. Missing school because of not feeling well	○	○	○	○	○
w. Missing school to go to the doctor or hospital	○	○	○	○	○

PEDS QL 4.0 Parent (13-18) © Copyright 1998 JW Varni, PhD. All rights reserved.
Not to be reproduced without permission

B. Your child's well-being and his/her size

Understanding the impact of your child's health and treatment (e.g. exercise, diet) on their day-to-day activities can help healthcare professionals provide better treatment recommendations for you and your child. For this reason, a weight-specific quality of life measure for parents of children with obesity was developed.

Instructions: The following questions are regarding your child's quality of life and your perceptions of how their weight/shape/size impacts their day to day activities. Please answer all the questions. There are no right or wrong answers. If you are unsure how to answer a particular question, please choose the response that seems to best fit your child's situation.

B.1. Has your child been on vacation, out of school, or had any major changes (e.g. moving, starting a new school) during the past month?

○ No ○ Yes (please explain) ______________________________

Please indicate how your child has been feeling within the past **MONTH** regarding their weight/shape/size by filling in the circle that best fits your child

During the past **month**, please tell us how often your child... (please fill in one circle on each row)	Never	Sometimes	Often	Always
a. Had difficulty participating in physical activities (e.g. sports) because of their weight/shape/size	○	○	○	○
b. Was teased by peers because of their weight/shape/size	○	○	○	○
c. Chose not to go to school because of their weight/shape/size	○	○	○	○
d. Felt sad because of their weight/shape/size	○	○	○	○
e. Had to make changes to surroundings (e.g. furniture, school desks) because of their weight/shape/size	○	○	○	○
f. Argued about when, what and how much to eat	○	○	○	○
g. Chose not to participate in gym/recess/physical education at school because of their weight/shape/size	○	○	○	○
h. Felt frustrated because of their weight/shape/size	○	○	○	○
i. Avoided dressing or undressing in front of others because of their weight/shape/size	○	○	○	○
j. Kept their body clean and fresh	○	○	○	○

During the past **month**, please tell us how often your child... (please fill in one circle on each row)	Never	Sometimes	Often	Always
k. Felt worried because of their weight/shape/size	○	○	○	○
l. Felt left out because of their weight/shape/size (e.g. no one talks or sits with them)	○	○	○	○
m. Felt mad because of their weight/shape/size	○	○	○	○
n. Was teased by others when physically active because of their weight/shape/size	○	○	○	○
o. Seen as having a good sense of humour	○	○	○	○
p. Felt concerned about their weight/shape/size	○	○	○	○
q. Perceived as healthy by others	○	○	○	○
r. Became upset at mealtimes (e.g. cried, fussed, argued)	○	○	○	○
t. Had difficulty keeping up with other children because of their weight/shape/size	○	○	○	○
u. Felt successful in daily activities	○	○	○	○
v. Became out of breath and had to slow down because of their weight/shape/size	○	○	○	○
w. Had low self esteem because of their weight/shape/size	○	○	○	○

For parents of children **14 years and older**:

During the past **month**, please tell us how often your child... (please fill in one circle on each row)	Never	Sometimes	Often	Always
x. Talked about difficulties dating due to their weight/shape/size	○	○	○	○
y. Preferred to spend time alone because of their weight/shape/size	○	○	○	○
z. Participated in hobbies/clubs (e.g. church group, school club, scouts/guides)	○	○	○	○
aa. Found it difficult to find a job/volunteer activity because of their weight/shape/size	○	○	○	○
bb. Worried about the future because of their weight/shape/size	○	○	○	○
cc. Attended extracurricular school activities (e.g. dances, sporting events, clubs, concerts)	○	○	○	○

Cincinnati Children's Hospital Medical Center © (Modi & Zeller, 2008)

C. How you have been feeling

Now. we would like to know a bit more about how **you** have been feeling. If you are worried about anything, be sure to talk about it with your doctor.

Remember the questions below are about you, **not** your child.

Now we'd like you to think about how **you** have been feeling during the **past month**. For each question, please fill in one circle that best describes how often you had this feeling.

During the **past month**, how often did **you** feel...

	**None** of the time	**A little** of the time	**Some** of the time	**Most** of the time	**All** of the time
**a.** ...tired out for no good reason	○	○	○	○	○
**b.** ...nervous	○	○	○	○	○
**c.** ... so nervous that nothing could calm you down	○	○	○	○	○
**d.** ... hopeless	○	○	○	○	○
**e.** ... restless or fidgety	○	○	○	○	○
**f.** ... so restless that you could not sit still	○	○	○	○	○
**g.** ... depressed	○	○	○	○	○
**h.** ... so depressed that nothing could cheer you up	○	○	○	○	○
**i.** ... that everything was an effort	○	○	○	○	○
**j.** ... worthless	○	○	○	○	○

K10 Kessler et al (2003)

Please check that you have answered all questions on both sides of each page.
Return to the researcher who is working with you today.

Thank you for your participation!

This page has been left blank intentionally.
