# Supplementary material for: New directions in childhood obesity research: how a comprehensive biorepository will allow better prediction of outcomes
Source: BMC Med Res Methodol. 2010 Oct 22;10:100. doi: 10.1186/1471-2288-10-100 (PMC2984501; doi:10.1186/1471-2288-10-100)
Supplement: Additional file 12 — L. COBRA Survey 2 Child 5-7yo.pdf. COBRA Survey 2 for children aged 5-7 years [file 1471-2288-10-100-S12.PDF]

6236283987

Participant code: | | | | | | | | | | | | | |

COBRA
Childhood Overweight BioRepository of Australia

Survey 2 (CHILD)

This form is for 5 to 7 year olds

Thank you for being in this study. This study is looking at the things that sometimes affect health problems for some overweight or obese children.

Before you see the doctors, we would like to get an idea about your general health and how well you feel. A researcher will help you with the questions on the next pages. You can talk to your parents or your doctor about the questions **after** you are finished.

This should take you about 15 minutes.

It is private, and your answers are confidential.

INSTRUCTIONS

1. Please answer by filling in the circles completely like this ●
2. If you make a mistake, put a cross through it, then fill in and draw a circle around the correct one.
3. Use a blue or black pen only.
4. There are no right or wrong answers. If you aren't sure, just give the best answer you can. You can also make a comment in the margin - it will be read!
5. Please remember to fill in the back of each page as well.

When you are done, please give this survey to the researcher working with you today.

Questions? Ring us (03) 9936 6512 or email us (mpowr@mcri.edu.au) any time

Thank you for taking part in COBRA

OFFICE USE ONLY

Date returned: ☐☐ / ☐☐ / ☐☐☐☐

Was survey completed before seeing clinicans? ○ No ○ Yes

Completed at: ○ RCH ○ Home ○ Other ____________

Murdoch Childrens Research Institute
Healthier Kids. Healthier Future.

The Royal Children's Hospital Melbourne

A. Your general health and well-being (interviewer administered)

Interviewer: I am going to ask you some questions about things that might be a problem for some children. I want to know how much of a problem any of these things might be for you.

If it is never a problem for you, point to the smiling face
If it is sometimes a problem for you, point to the middle face
If it is almost always problem for you, point to the frowning face

I will read each question. Point to the pictures to show me how much of a problem it is for you. Let's try a practice one first.

	Never ☺	Sometimes 😐	Almost always ☹
Is it hard for you to click your fingers?	○	○	○

Think about how you have been for the last few weeks. Please listen carefully to each sentence and tell me how much of a problem this is for you.

**Physical Functioning (problems with...)**	Never ☺	Sometimes 😐	Almost always ☹
a. It is hard for you to walk?	○	○	○
b. It is hard for you to run?	○	○	○
c. It is hard for you to play sport or do exercise?	○	○	○
d. It is hard for you to pick up big things?	○	○	○
e. It is hard for you to have a bath or shower?	○	○	○
f. It is hard for you to help around the house (like pick up your toys)?	○	○	○
g. Do you get aches or pains? Where? ______________________	○	○	○
h. Do you ever feel too tired to play?	○	○	○

**Emotional Functioning (problems with...)**	Never ☺	Sometimes 😐	Almost always ☹
i. Do you feel scared?	○	○	○
j. Do you feel sad?	○	○	○
k. Do you feel angry?	○	○	○
l. Do you have trouble sleeping?	○	○	○
m. Do you worry about what will happen to you?	○	○	○

Social Functioning (problems with...)	Never ☺	Sometimes 😐	Almost always ☹
n. Is it hard for you to get along with other kids?	○	○	○
o. Do other kids say they do not want to play with you?	○	○	○
p. Do other kids tease you?	○	○	○
q. Can other kids your age do things that you cannot do?	○	○	○
r. Is it hard for you to keep up when you play with other kids?	○	○	○

School/Preschool Functioning (problems with...)	Never ☺	Sometimes 😐	Almost always ☹
s. Is it hard for you to pay attention at school/preschool?	○	○	○
t. Do you forget things?	○	○	○
u. Is it hard for you to keep up with work at school/preschool?	○	○	○
v. Are you away from school/preschool because you feel sick?	○	○	○
w. Are you away from school/preschool because you have to go to the doctor's or hospital?	○	○	○

PEDS QL 4.0 (C5-7) © Copyright 1998 JW Varni, PhD. All rights reserved.
Not to be reproduced without permission

B. Your well-being & your size (interviewer administered)

Now you are going to answer some questions, but first we want to go over the different answer choices with you.

If I asked you to pick ALL of the circle, which would you pick? (Answer = 3). If I asked you to pick **A lot** of the circle, which would you pick? (Answer = 2) If I asked you to pick **A little** of the circle, which would you pick? (Answer = 1). If I asked you to pick **None** of the circle, which would you pick? (Answer = 0).

We are going to be asking you some questions about some of the things that you think and feel . There are no right or wrong answers. For each question I ask you, you are going to look at the choices below and choose an answer. If you are not sure about your answer, just pick the one that you think is best for you.

EXAMPLE: A library has books.
Is that "none of the time," "a little," "a lot," or "all the time"?

EXAMPLE: Dogs can fly.
Is that "none of the time," "a little," "a lot," or "all the time"?

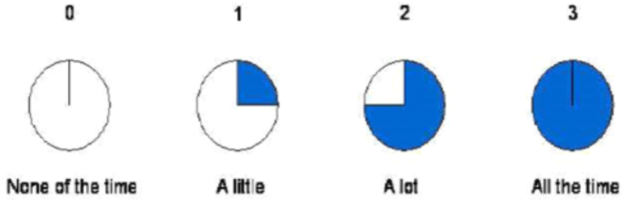

Remember to fill the circles like this ●

During the past month, please tell us how much you... (please fill in one circle on each row)	None	A little of the time	A lot of the time	All the time
a. Were teased by other kids because of your size	○	○	○	○
b. Felt sad because of your size	○	○	○	○
c. Were told you are healthy or growing well	○	○	○	○
d. Felt mad because of your size	○	○	○	○
e. Felt left out because of your size (e.g. no one talks or sits with you)	○	○	○	○
f. Found it hard to swing, climb, skip, bounce a ball, or jump rope because of your size	○	○	○	○
g. Like yourself because of your size	○	○	○	○
h. Stood up for or helped other kids because of your size	○	○	○	○
i. Felt frustrated because of your size	○	○	○	○
j. Felt worried because of your size	○	○	○	○
k.Chose not to go to school because of your size	○	○	○	○
l. Had problems fitting into your desk at school because of your size	○	○	○	○
m. Felt happy because of your size	○	○	○	○
n. Were picked first for recess or gym because of your size	○	○	○	○
o. Were teased by other kids when physically active (e.g. move your body) because of your size	○	○	○	○
p. Felt you have a good sense of humour	○	○	○	○
q. Did not want to go to the swimming pool or park because of your size	○	○	○	○
r. Felt uncomfortable sleeping at a friend's house because of your size	○	○	○	○
t. Got upset at mealtimes (e.g. cried, fussed, argued)	○	○	○	○
u. Found it hard to keep up with other kids because your size	○	○	○	○
v. Got out of breath and had to slow down because of your size	○	○	○	○
w. Chose not to participate in gym or recess at school because of your size	○	○	○	○

Cincinnati Children's Hospital Medical Center © (Zeller & Modi, 2009)

Please check that you have answered all questions on both sides of each page.
Return to the researcher who is working with you today.

Thank you for your participation!
