# Supplementary material for: New directions in childhood obesity research: how a comprehensive biorepository will allow better prediction of outcomes
Source: BMC Med Res Methodol. 2010 Oct 22;10:100. doi: 10.1186/1471-2288-10-100 (PMC2984501; doi:10.1186/1471-2288-10-100)
Supplement: Additional file 13 — M. COBRA Survey 2 Youth 8-12yo.pdf. COBRA Survey 2 for youth aged 8-12 years [file 1471-2288-10-100-S13.PDF]

7028454695

Participant code: [ ][ ][ ][ ][ ][ ][ ][ ][ ][ ][ ][ ][ ]

COBRA
Childhood Overweight BioRepository of Australia

Survey 2 (YOUTH)

This form is for 8 to 12 year olds

Thank you for being in this study. This study is looking at the things that may affect health problems for some overweight or obese children.

Before you see the doctors, we would like to get an idea about your general health and how well you feel. Please answer the questions on the next pages. Please answer them by yourself (you can talk to your parents or your doctor about the questions **after** you have finished). A researcher is here to help you if you have any questions.
This should take you about 10 minutes.

It is private, and your answers are confidential.

INSTRUCTIONS

1. Please answer by filling in the circles completely like this ●
2. If you make a mistake, put a cross through it, then fill in and draw a circle around the correct one.
3. Use a blue or black pen only.
4. There are no right or wrong answers. If you aren't sure, just give the best answer you can. You can also make a comment in the margin - it will be read!
5. Please remember to fill in the back of each page as well.

When you are done, please give this survey to the researcher working with you today.

Questions? Ring us (03) 9936 6512 or email us (mpowr@mcri.edu.au) any time

Thank you for taking part in COBRA

OFFICE USE ONLY

Date returned: [ ][ ] / [ ][ ] / [ ][ ][ ][ ]

Was survey completed before seeing clinicans? ○ No ○ Yes

Completed at: ○ RCH ○ Home ○ Other ____________

Murdoch Childrens Research Institute — Healthier Kids. Healthier Future.
The Royal Children's Hospital Melbourne

A. Your health and wellbeing (interviewer assisted)

Below is a list of things that might be a problem for **you**. Please tell us **how much of a problem** each one has been for you during the **past ONE month** by filling in the circle under the colums if the problem is:

- **never** a problem
- **almost never** a problem
- **sometimes** a problem
- **often** a problem
- **almost always** a problem

There are no right or wrong answers. If you do not understand a question, please ask for help.
In the **LAST ONE MONTH**, how much of a problem has this been for you...

**About my health and activities (problems with...)**	**Never**	**Almost Never**	**Some-times**	**Often**	**Almost Always**
a. It is difficult for me to walk a few houses from home (about 100 metres)	○	○	○	○	○
b. It is difficult for me to run	○	○	○	○	○
c. It is difficult for me to play sport or do exercise	○	○	○	○	○
d. It is difficult for me to lift something heavy	○	○	○	○	○
e. It is difficult for me to have a bath or shower by myself	○	○	○	○	○
f. It is difficult for me to help around the house	○	○	○	○	○
g. I get aches and pains	○	○	○	○	○
h. I have low energy	○	○	○	○	○

**About my feelings (problems with...)**	**Never**	**Almost Never**	**Some-times**	**Often**	**Almost Always**
i. I feel afraid or scared	○	○	○	○	○
j. I feel sad	○	○	○	○	○
k. I feel angry	○	○	○	○	○
l. I have trouble sleeping	○	○	○	○	○
m. I worry about what will happen to me	○	○	○	○	○

**How I get along with others (problems with...)**	**Never**	**Almost Never**	**Some-times**	**Often**	**Almost Always**
n. I have trouble getting along with other kids	○	○	○	○	○
o. Other kids do not want to be my friend	○	○	○	○	○
p. Other kids tease me	○	○	○	○	○
q. I cannot do things that other kids my age can do	○	○	○	○	○
r. It is hard to keeping up when I play with other kids	○	○	○	○	○

**About school (problems with...)**	**Never**	**Almost Never**	**Some-times**	**Often**	**Almost Always**
s. It is hard to pay attention in class	○	○	○	○	○
t. I forget things	○	○	○	○	○
u. I have trouble keeping up with my schoolwork	○	○	○	○	○
v. I miss school because I feel sick	○	○	○	○	○
w. I am away from school to go to the doctor or hospital	○	○	○	○	○

PEDS QL 4.0 (C 8-12) © Copyright 1998 JW Varni, PhD. All rights reserved.
Not to be reproduced without permission

B. Your feelings & your size (interviewer assisted)

Now you are going to answer some questions, but first we want to go over the different answer choices with you.

If I asked you to pick ALL of the circle, which would you pick? (Answer = 3). If I asked you to pick **A lot** of the circle, which would you pick? (Answer = 2) If I asked you to pick **A little** of the circle, which would you pick? (Answer = 1). If I asked you to pick **None** of the circle, which would you pick? (Answer = 0).

We are going to be asking you some questions about some of the things that you think and feel . There are no right or wrong answers. For each question I ask you, you are going to look at the choices below and choose an answer. If you are not sure about your answer, just pick the one that you think is best for you.

EXAMPLE: A library has books.
Is that "none of the time," "a little," "a lot," or "all the time"?

EXAMPLE: Dogs can fly.
Is that "none of the time," "a little," "a lot," or "all the time"?

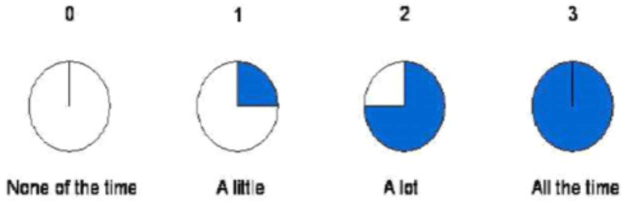

During the past month, please tell us how much you... (please fill in one circle on each row)	**None**	**A little of the time**	**A lot of the time**	**All the time**
a. Were teased by other kids because of your size	○	○	○	○
b. Felt sad because of your size	○	○	○	○
c. Were told you are healthy or growing well	○	○	○	○
d. Felt mad because of your size	○	○	○	○
e. Felt left out because of your size (e.g. no one talks or sits with you)	○	○	○	○
f. Found it hard to swing, climb, skip, bounce a ball, or jump rope because of your size	○	○	○	○
g. Like yourself because of your size	○	○	○	○
h. Stood up for or helped other kids because of your size	○	○	○	○
i. Felt frustrated because of your size	○	○	○	○
j. Felt worried because of your size	○	○	○	○
k.Chose not to go to school because of your size	○	○	○	○
l. Had problems fitting into your desk at school because of your size	○	○	○	○

During the past month, please tell us how much you... (please fill in one circle on each row)	**None**	**A little of the time**	**A lot of the time**	**All the time**
m. Felt happy because of your size	○	○	○	○
n. Were picked first for recess or gym because of your size	○	○	○	○
o. Were teased by other kids when physically active (e.g. move your body) because of your size	○	○	○	○
p. Felt you have a good sense of humour	○	○	○	○
q. Did not want to go to the swimming pool or park because of your size	○	○	○	○
r. Felt uncomfortable sleeping at a friend's house because of your size	○	○	○	○
t. Got upset at mealtimes (e.g. cried, fussed, argued)	○	○	○	○
u. Found it hard to keep up with other kids because your size	○	○	○	○
v. Got out of breath and had to slow down because of your size	○	○	○	○
w. Chose not to participate in gym or recess at school because of your size	○	○	○	○

Cincinnati Children's Hospital Medical Center © (Zeller & Modi, 2009)

Please check that you have answered all questions on both sides of each page.
Return to the researcher who is working with you today.

Thank you for your participation!
